# The Role of the Digital Workplace in Enhancing Productive Organizational Energy: An Analytical Study at the University of Fallujah

**DOI:** 10.12688/f1000research.174088.1

**Published:** 2026-02-11

**Authors:** Yasser Lateef Khalaf, Amna Laith Alhashimi

**Affiliations:** 1 public administration, University of Fallujah, Al-Fallujah, Al Anbar Governorate, Iraq; 2 public administration, University of Fallujah, Al-Fallujah, Al Anbar Governorate, Iraq

**Keywords:** Digital workplace, productive organizational energy, University of Fallujah.

## Abstract

**Background:**

Although digital workplaces are increasingly being adopted across academic institutions, many universities still perceive them merely as technological tools rather than as integrated socio-organizational systems that foster productive organizational energy. This study addresses this gap by examining the effect of digital workplace dimensions—digital space, aesthetics, capabilities, and intelligence—on productive organizational energy at the University of Fallujah.

**Methods:**

Using a descriptive-analytical approach, data from 290 respondents were analyzed using SPSS v.28 and Smart PLS v.4, employing confirmatory factor analysis and multiple regression.

**Results:**

The overall digital workplace demonstrated a strong positive influence on productive organizational energy (β = 0.892, R ^2^ = 0.75, F = 865.962, P < 0.001). Among these dimensions, digital intelligence exerted the highest impact (β = 0.819, R ^2^ = 0.69), followed by digital capabilities (β = 0.775, R ^2^ = 0.67), digital space (β = 0.651, R ^2^ = 0.59), and digital aesthetics (β = 0.665, R ^2^ = 0.48). These findings confirm that smart technologies and analytics significantly enhance emotional, cognitive, and behavioral energy across staff, thereby improving motivation and efficiency.

**Conclusions:**

Theoretically, this research extends the digital transformation literature by linking digital workplace constructs to organizational energy, underscoring the necessity of strengthening digital intelligence and employee capabilities to advance institutional performance.

## 1. Introduction

The digital workplace concept has transformed immensely over the last 20 years, becoming a key cornerstone of business transformation. A digital Workplace is characterized as a combined set of devices, applications, and digital networks that allow employees to perform their tasks flexibly from anywhere bringing  productivity and organizational agility ( de Moraes et al., 2024; Ujwary-Gil & Godlewska-Dzioboń, 2022). In the context of higher education institutions, digital transformation is a key factor for optimizing operations and communication, as well as service quality, and plays a critical role in both organizational innovation and user satisfaction ( Mishra et al., 2023; Too et al., 2025; Al-Rawi et al., 2021). Researchers have conceptualized the digital workplace as a vibrant ecology consisting of technological, social and organizational aspects that influence interaction and collaboration in media-saturated environments ( Chawla & Goyal, 2022; Marsh et al., 2024). The latest technological developments have changed the nature of traditional work designs, leading to enhanced remote collaboration, cross-functional integration, and employee performance and quality of life ( Meske & Junglas, 2021; Nesindande et al., 2024).

The current literature indicates that the digital workplace enhances flexibility, connectivity, and inclusiveness through remote access, collaborative platforms, and evidence-based intelligence ( Attaran et al., 2019; Kaur et al., 2025; Rakovic et al., 2022). By doing so, such digital spaces advance employees’ engagement through collaboration and ongoing learning, comparing performance with one another. and provides analytical tools to managers ( Eckhardt et al., 2019; Hoert et al., 2018; Husien et al., 2018). Nevertheless, the application of digital workplaces is uneven throughout industries, especially in academia, where there is a tendency to characterize digitalization only as tool adoption and not from an organizational or behavioral perspective ( Zimmer et al., 2023). consequently, the potential benefits of digital technologies for organizational effectiveness and innovation are underexploited ( Khin & Ho, 2019; Usai et al., 2021).

Additionally, the notion of productive organizational energy has been identified as a crucial aspect of institutional vitality and effectiveness ( Abualhamael, 2017; Alexiou et al., 2019; Cole et al., 2005; Obeng et al., 2021; Husien et al., 2019). This refers to the set of emotional, cognitive, and behavioral resources through which creativity, focus, and resilience contribute to achieving organizational objectives ( Baker, 2019; Gabardo-Martins, 2022). Prior research indicates that high levels of OE strengthen job satisfaction, commitment, and collaborative performance, thereby fostering institutional competitive advantage ( Cuff & Barkhuizen, 2014; Vogel et al., 2022). Notwithstanding these insights, there is limited_ empirical evidence associating the dimensions of the digital workplace—namely, digital space, capabilities, aesthetics, and intelligence – to the activation of productive organizational energy, especially in higher education institutions in developing countries ( Kelder et al., 2025; White, 2012; Al-Sabaawe et al., 2020).

This research bridges _this gap and explores how digital workplaces influence the productive organizational energy of the University of Fallujah. This study seeks to broaden the theoretical perspectives of digital workplaces by intertwining them with the theory of the organizational energy field, to understand how digital transformation impacts employees’ emotional, cognitive, and behavioral engagement. The empirical research in this paper has both theoretical and application dimensions for the evolution of digital transformation literature as well as practice for institutions of higher education regarding how they could best design their digital space to achieve motivation, collaboration, and overall organizational performance.

## 2. Methods

Research Methodology: The researchers in their study relied on a descriptive analytical method, which is considered appropriate for providing a clear and accurate picture of the phenomenon of the research problem. This is because it studies the characteristics and forms of events and phenomena and combines more than one approach at the same time, represented by observations, questionnaires, and personal interviews, which directly access information.

### 2.1 Sample characterization

2.1.1 Gender

Table 1 displays the frequency distribution of the sample members by gender at the University of Fallujah. There were 290 sample members, and most of the sample (80.3%) was male (233 members). Only 57 participants were female (19.7%). Female involvement in the study was much lower than male participation, which may be due to the university’s largely male functional or academic organization.

**Table 1.** Frequency distribution by gender.

Gender	Frequency	Percentage (%)
Male	233	80.3
Female	57	19.7
Total	290	100.0

2.1.2 Age

Table 2 shows the frequency distribution of the sample members according to the age group at the University of Fallujah.

**Table 2.** The distribution by age.

Age group	Frequency	Percentage (%)
25-30	55	19.0
31-35	45	15.5
36-40	46	15.9
41-45	45	15.5
46-50	43	14.8
51-55	34	11.7
Higher than 55	22	7.6
Total	290	100

2.1.3 Academic achievement

Table 3 shows the frequency distribution of the sample members according to their academic achievements at the University of Fallujah.

**Table 3.** The distribution based on level of academic achievement.

Academic achievement	Frequency	Percentage (%)
Bachelor's Degree	87	30.0
Higher Diploma	48	16.6
Master's Degree	77	26.6
Doctorate	78	26.9
Total	290	100

2.1.4 Years of service

Table 4 presents the frequency distribution of the respondents according to their years of service at the University of Fallujah.

**Table 4.** The frequencies by years of service.

Years of service	Frequency	Percentage (%)
Less than 5 years	55	19.0
6–10 years	98	33.8
11–15 years	34	11.7
16–20 years	82	28.3
More than 20 years	21	7.2
**Total**	**290**	**100**

2.1.5 Administrative position

Table 5 shows the frequency distribution of the sample members according to their administrative positions at the University of Fallujah. The research sample was a balanced representation of all administrative and academic levels, with a clear dominance of teaching staff. This lends the study’s findings to academic depth and provides a realistic reflection of the university’s organizational structure.

**Table 5.** The frequency distribution by administrative position.

Administrative position	Frequency	Percentage (%)
Employee	45	15.5
Faculty Member	105	36.2
Division Head	56	19.3
Department Head	76	26.2
Dean	4	1.4
Assistant Dean	4	1.4
**Total**	**290**	**100**

2.1.6 Academic rank

Table 6 presents the frequency distribution of the respondents according to their academic rank at the University of Fallujah. The sample tends towards higher academic levels, as the categories (Professor, Assistant Professor, and Assistant Lecturer) constitute more than two-thirds of the sample, which gives the study results cognitive depth and scientific rigor stemming from advanced academic experiences within the university.

**Table 6.** The frequency distribution by academic rank.

Academic rank	Frequency	Percentage (%)
None	46	15.9
Professor	78	26.9
Associate Professor	68	23.4
Lecturer	40	13.8
Assistant Lecturer	58	20.0
**Total**	**290**	**100**

## 3. Results and discussion

### 3.1 Response rate

To ensure fulfillment of the study’s requirements, the researcher distributed (300) questionnaires to a random sample of affiliates at Fallujah University. 290 were retrieved and validated for statistical analysis, as shown in Table 7.

**Table 7.** The number of questionnaires distributed.

Status	Number of distributed questionnaires	Number of unreturned questionnaires	Number of valid questionnaires for analysis
Number	300	10	290
Percentage	100	3.333	96.67

### 3.2 Results of the descriptive analysis

The conclusions regarding the responses of the sample were based on reviewing the information and analyzing it using statistical methods. The findings will be discussed in light of the current research axes, and an effort will be made to interpret these relationships and findings.

3.2.1 Digital workplace independent variable

According to Table 8, the digital workplace variable featured relative importance order (first), overall arithmetic mean (3.350), standard deviation (PCS = 0.805), coefficient of variation (24.04%), and medium evaluation level. This therefore indicates that the productive organizational ‘energy’ environment is keen to embrace digital workplace technologies ( Marsh et al., 2024). However, this level is not of strong adoption, but moderate inclination towards digital transformation, which may be due to technical or organizational barriers such as limited technical resources and institutional unawareness ( Mishra et al., 2023). The highest mean of (3.424), SD = 0.845, and CV = (24.67%) were reported for digital intelligence and attained a level classification of (good). This indicates an interest in using intelligent technologies to analyze data and make decisions, which can be considered as a progress of awareness within the university about the role of smart digital solutions, although it is not experienced at the same level by all administrative units. Second, digital aesthetics had a median score of 3.386, standard deviation of 0.870, and coefficient of variation of 25.70%, all at (medium) level of rating. This indicates a kind of tension of the attractive power of digital systems to look, as well as being interacted with by users. These efforts, however, need to be better infused into the interface design and user experience for a more meaningful impact on increasing work efficiency. Meanwhile, the digital (3.294), std. Focused capabilities were third with 0.878 std and a variation coefficient of (26.65%) therefore, an evaluation tier of (Medium) levels despite over the actionable level these capabilities should be posed in evaluating as they have, on average, some high impact and important (Medium) level, but also with possible higher potential development. This represents a modest level of digital proficiency among employees ( Hoert et al., 2018; Meske & Junglas, 2021). However, it also means that it is important to extend the training and skills programmed to improve the technical efficiency necessary to handle digitalization. Regarding digital, it obtained the lowest (average = 3.297, SD = 0.983, and CoV = 29.80%), with a rating level of (medium). This could mean that digital shared workspaces are not yet widespread and/or that there might be some limitations in terms of infrastructure or the availability of fully integrated digital systems. From this analysis, we can conclude that the measurement findings confirm the digital workplace variable at a medium scale biased towards positivity, with a higher advantage of pillar maturity appearing in digital intelligence rather than the others, as well as benchmarking scale by taking more development on training, interaction design, and digital infrastructure to establish an efficient and sustainable environment for work ( Marsh et al., 2024).

1. Productive Organizational Energy Dependent Variable

**Table 8.** Descriptive statistics for research variables and dimensions.

Research variables and dimensions	M	S	CV	Rank	Answer trend
Digital Space	3.297	0.983	29.80	4	Average
Digital Beauty	3.386	0.870	25.70	2	Average
Digital Capabilities	3.294	0.878	26.65	3	Average
Digital Intelligence	3.424	0.845	24.67	1	Good
Digital Workplace	3.350	0.805	24.04	First	Average
Emotional Energy	3.487	0.769	22.04	1	Good
Cognitive Energy	3.376	0.921	27.28	2	Average
Behavioral Energy	3.349	0.989	29.54	3	Average
Organizational Energy	3.404	0.829	24.35	Second	Average

Findings ( Table 8) revealed that the productive organization energy variable came at the second order regarding relative importance with a total arithmetic mean and a standard deviation of (3.404), and (0.829), respectively, equal to (.3435), a medium evaluation level. This implies a positive interest in the utilization of effective organizational energy practices within the work organization of universities. However, this level does not indicate high organizational vitality, but simply a situation of lack of negative as well as the presence of a low amount of positive interaction that can be enhanced by creating a motivating work environment and concentration on both employees’ behavioral and emotional energy sources ( Hoert et al., 2018; Meske & Junglas, 2021). Based on the analysis of dimensions, emotional energy held first place with an average (3.487), standard deviation (0.769) and, coefficient of variation (22.04%), and evaluated level. This is evidence of the good state of enthusiasm and satisfaction enjoyed by staff members, while working with vitality and activity, which means the availability of an inspiring campus atmosphere that reflects a spirit of pride and sense of belonging to the university. Cognitive energy arrived in second with an arithmetic mean of (3.376), a standard deviation of (0.921), and a coefficient of variation of (27.28%) along with a medium evaluation level. This is indicative of relatively high cognitive readiness among employees to share knowledge and actively look for opportunities to continuously develop themselves ( Hoert et al., 2018). However, the high coefficient of variation means that there is still a disproportion in this area among individuals, so we must reinforce the culture of organizational learning and intellectual participation. On the other hand, behavioral energy ranked third with (3.349) as an average difference, (0.989) as a standard deviation, and (29.54%) coefficient of variation for the previous evaluation level attempting to measure this variable means that the nature of its application in practical work still needs to be improved, where average measures indicate that this level lacks initiative or practical activity, especially since there is a discrepancy in efforts between employees. To instill a spirit of dedication and willpower, it calls for incessant encouragement, guidance, and organizational support. The analysis findings suggest that the organizational productive energy variable is positioned at an intermediate level of intensity, based on emotional energy, which translates into the main motivating source for employees to outperform, with cognitive and behavioral energies requiring more long-term development programs of motivation-enhancement in support of new forms of organizational learning and effective behavior within the university work context ( Hoert et al., 2018).

### 3.3 Confirmatory construct validity

3.3.1 Confirmatory factor analysis for digital workplace

**Figure 1.**
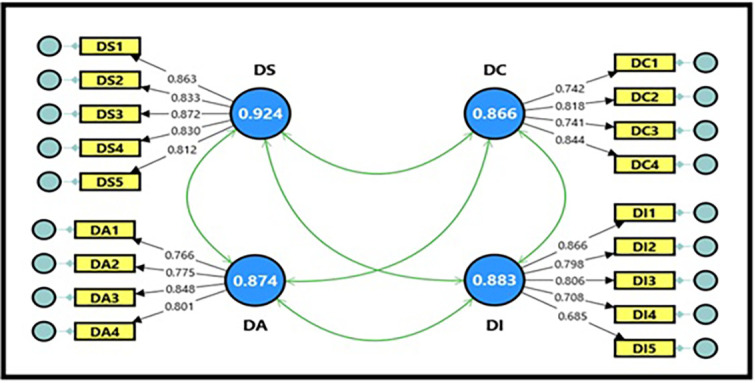
Confirmatory factor analysis of digital workplace.

As an independent variable, the digital workplace model consisted of four main dimensions: (digital space, digital aesthetics, digital capabilities, and digital intelligence) with 18 questions, as demonstrated in Figure 1. Table 9 shows the extracted fit quality indicators, which were within the required standards for model acceptance. All composite reliability (CR) magnitudes for the digital workplace variable were within acceptable limits, reaching (0.874, 0.867, 0.886, and 0.924), which is a good indicator and denotes the reliability of the scale, reflecting a high internal consistency of the scale’s dimensions ( Marsh et al., 2024). It is also clear from the Cronbach’s alpha coefficient magnitudes, which reached (0.874, 0.866, 0.883, and 0.924), that they are greater than (0.70), indicating high reliability between items for each dimension of the model. The findings demonstrated that all average variance extracted (AVE) magnitudes for the digital workplace variable were acceptable, ranging between (0.637, 0.621, 0.602, and 0.710), which is greater than the standard magnitude (0.50), indicating that the sub-dimensions contribute significantly to explaining the total variance of the digital workplace variable; thus, the model is considered more reliable in interpreting the relationships between its dimensions.

**Table 9.** Fit quality indicators for digital workplace.

Dimensions	Cronbach’s alpha (standardized)	Cronbach’s alpha (unstandardized)	Composite reliability (rho_c)	Average variance extracted (AVE)
DA	0.874	0.873	0.874	0.637
DC	0.866	0.865	0.867	0.621
DI	0.883	0.883	0.886	0.602
DS	0.924	0.924	0.924	0.710

Table 10 shows the estimation magnitudes, which ranged between (0.685 and 0.872) for all items representing the dimensions of the digital workplace variable. All items have high factor loading coefficients and exceed the acceptable minimum limit of (0.50), indicating that all items have a clear impact on their dimensions. It is also evident from the T-statistic magnitudes, which ranged between (12.288 and 19.761), that all of them are greater than the tabular magnitude of (1.984) at a significance level of (0.05), which is a sufficient indicator for adopting the model in its final form for subsequent analyses, and reflects the strength of the relationships between the items and the constituent dimensions of the digital workplace variable ( Marsh et al., 2024).

**Table 10.** Estimates for the dimensions of the digital workplace variable.

Paragraphs	Parameter estimates	Standard errors	T magnitudes	P magnitudes
DA1 <- DA	0.766	n/a	n/a	n/a
DA2 <- DA	0.775	0.082	13.514	0.000
DA3 <- DA	0.848	0.076	14.399	0.000
DA4 <- DA	0.801	0.072	14.044	0.000
DC1 <- DC	0.742	n/a	n/a	n/a
DC2 <- DC	0.818	0.084	14.031	0.000
DC3 <- DC	0.741	0.086	12.288	0.000
DC4 <- DC	0.844	0.078	14.277	0.000
DI1 <- DI	0.866	n/a	n/a	n/a
DI2 <- DI	0.798	0.053	17.029	0.000
DI3 <- DI	0.806	0.051	16.660	0.000
DI4 <- DI	0.708	0.051	13.568	0.000
DI5 <- DI	0.685	0.053	13.035	0.000
DS1 <- DS	0.863	n/a	n/a	n/a
DS2 <- DS	0.833	0.051	18.679	0.000
DS3 <- DS	0.872	0.052	19.761	0.000
DS4 <- DS	0.830	0.055	17.876	0.000
DS5 <- DS	0.812	0.055	17.397	0.000

3.3.2 Confirmatory factor analysis of productive organizational energy

**Figure 2.**
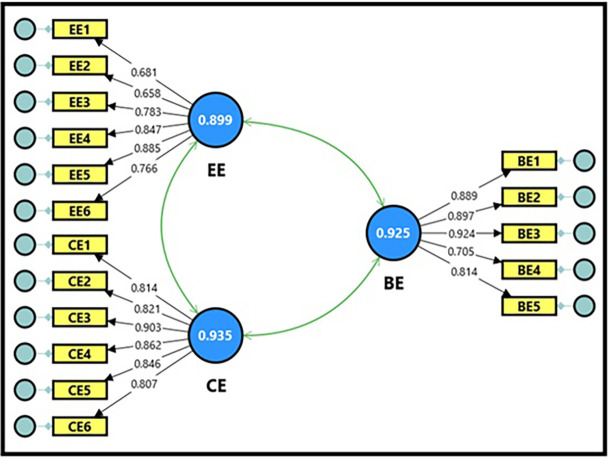
Confirmatory factor analysis of productive organizational energy.

The productive organizational energy model, as a variable, consists of three basic dimensions: behavioral, cognitive, and emotional energy, with (17) questions, as demonstrated in Figure 2. Table 11 shows the extracted fit quality indicators, which were within the required standards for model acceptance. The composite reliability (CR) magnitudes for the productive organizational energy variable were all within acceptable limits, reaching (0.929, 0.937, and 0.899), which is a good indicator and denotes the reliability of the scale, reflecting a high internal consistency of the scale’s dimensions. It is also clear from the Cronbach’s alpha magnitudes, which were (0.925, 0.935, and 0.899), which were greater than (0.70), indicating high reliability between the items for each dimension of the model. The findings demonstrated that the average variance extracted (AVE) magnitudes for the productive organizational energy variable were all acceptable, ranging between (0.599 and 0.722), which is greater than the standard magnitude (0.50), indicating that the sub-dimensions contribute significantly to explaining the total variance of the productive organizational energy variable; thus, the model is considered more reliable for interpreting the relationships between its dimensions.

**Table 11.** Fit quality indicators for productive organizational energy.

Dimensions	Cronbach’s alpha (standardized)	Cronbach’s alpha (unstandardized)	Composite reliability (rho_c)	Average variance extracted (AVE)
BE	0.925	0.925	0.929	0.722
CE	0.935	0.935	0.937	0.711
EE	0.899	0.898	0.899	0.599

Table 12 demonstrates the factor loading magnitudes, which ranged between (0.658 and 0.924) for all items comprising the dimensions of the productive organizational energy variable. All items were influential and had high factor loading coefficients exceeding the minimum acceptable limit of (0.50), reflecting the quality of their representation of their dimensions. It is also clear from the T-statistic magnitudes, which ranged between (10.592 and 24.110), that they were all greater than the tabular magnitude of (1.984) at a significance level of (0.05), which is considered a sufficient indicator for adopting the model in its final form in subsequent analyses, and reflects the strength of the correlation between the items and the dimensions comprising the productive organizational energy variable.

**Table 12.** Estimates for the dimensions of the productive organizational energy variable.

Items	Parameter estimates	T magnitudes	P magnitudes
BE1 <- BE	0.889	n/a	n/a
BE2 <- BE	0.897	22.742	0.000
BE3 <- BE	0.924	24.110	0.000
BE4 <- BE	0.705	14.511	0.000
BE5 <- BE	0.814	18.605	0.000
CE1 <- CE	0.814	n/a	n/a
CE2 <- CE	0.821	16.517	0.000
CE3 <- CE	0.903	18.980	0.000
CE4 <- CE	0.862	17.677	0.000
CE5 <- CE	0.846	17.356	0.000
CE6 <- CE	0.807	16.041	0.000
EE1 <- EE	0.681	n/a	n/a
EE2 <- EE	0.658	10.592	0.000
EE3 <- EE	0.783	12.307	0.000
EE4 <- EE	0.847	12.976	0.000
EE5 <- EE	0.885	13.338	0.000
EE6 <- EE	0.766	11.752	0.000

### 3.4 Testing research hypotheses

Testing the hypothesis between the dimensions of the (digital workplace) variable in the (productive organizational energy) variable is, shown in Table 13.

**Table 13.** Analysis of the impact between digital workplace dimensions on productive organizational energy.

Dependent variable	Sig	F	R ^2^Adj	(R ^2^)	R	t	Dimensions of the digital workplace		
**Organizational Productive Energy**	**0.000**	422.364	0.593	0.595	0.771	11.563	1.259	**α**	Digital Space
						20.552	0.651	**β**	
	**0.000**	273.197	0.485	0.487	0.698	8.205	1.153	**α**	Digital Beauty
						16.529	0.665	**β**	
	**0.000**	594.552	0.673	0.674	0.821	7.847	0.85	**α**	Digital Capabilities
						24.383	0.775	**β**	
	**0.000**	662.59	0.696	0.697	0.835	5.336	0.599	**α**	Digital Intelligence
						25.741	0.819	**β**	
	**0.000**	865.962	0.75	0.75	0.866	3.989	0.416	**α**	Digital Workplace
						29.427	0.892	**β**	

3.4.1 The first main hypothesis

**Figure 3.**
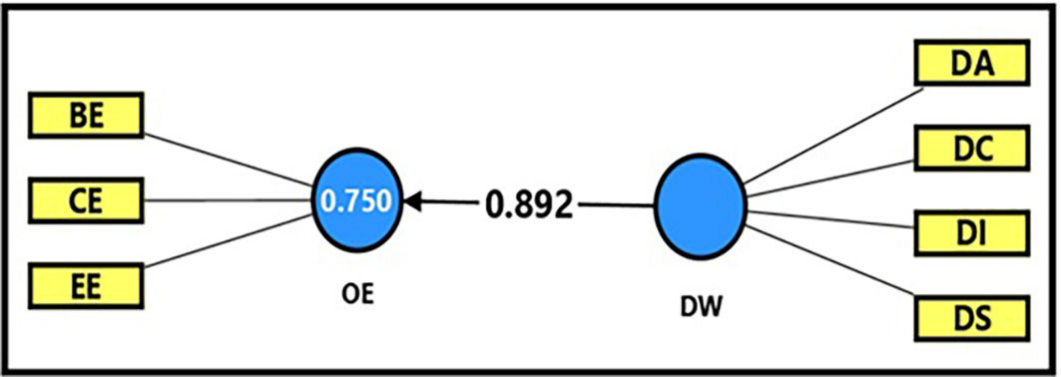
Analysis of the impact of the digital workplace on productive organizational energy.

It is clear from Table 13 and Figure 3 that the extracted (F) magnitude between the digital workplace and productive organizational energy was (865.962), which is greater than the tabular (F) magnitude of (3.94) at a significance level of (0.05). This result provides sufficient support for accepting the alternative hypothesis, which states that the digital workplace has a statistically significant impact on productive organizational energy. This indicates that the digital workplace has a strong significant impact on enhancing productive organizational energy, as it was able to explain (75%) of the changes occurring in productive organizational energy. In addition, the extracted (t) magnitude for the digital workplace variable was (29.427), which was greater than the tabular magnitude (1.984) at a significance level of (0.05), indicating the significance of (β) for the digital workplace variable. It is clear from the magnitude (β = 0.892) that an increase in the digital workplace by one unit leads to an increase in productive organizational energy by (89%), reflecting the strength of the direct impact and the high level of correlation between the two variables, This confirms that the digital work environment is one of the most prominent factors influencing the activation of productive organizational energies and improving institutional performance.

3.4.2 Testing sub-hypotheses for digital workplace dimensions in productive organizational energy

*3.4.2.1 First sub-hypothesis*

**Figure 4.**
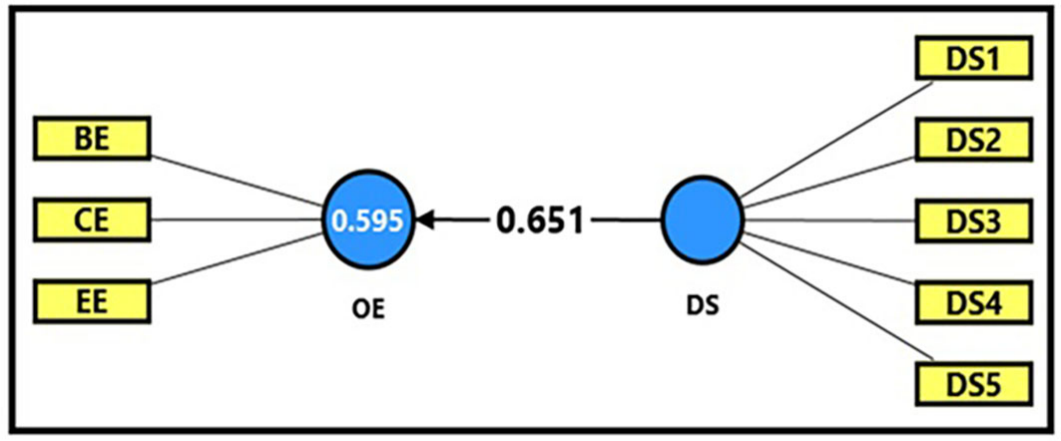
Analysis of the impact between the digital space dimension and productive organizational energy.

Table 13 and Figure 4 demonstrate the extracted (F) magnitude between digital space and productive organizational energy, which recorded (422.364), greater than the tabular (F) magnitude of (3.94) at a significance level of (0.05). This result provides sufficient support for the alternative hypothesis that digital space has a statistically significant impact on productive organizational energy. This indicates that digital space has a clear and significant impact on activating productive organizational energy, as it was able to explain (59%) of the changes occurring in it. The extracted (t) magnitude for the digital space variable was also recorded (20.552), which was greater than the tabular magnitude (1.984) at a significance level of (0.05), indicating the significance of (β) for the digital space variable. It is clear from the magnitude (β = 0.651) that an increase in digital space by one unit leads to an increase in productive organizational energy by (65%), which reflects the importance of enhancing a shared digital work environment to improve enthusiasm and commitment levels within academic institutions.

*3.4.2.2 Second sub-hypothesis*

**Figure 5.**
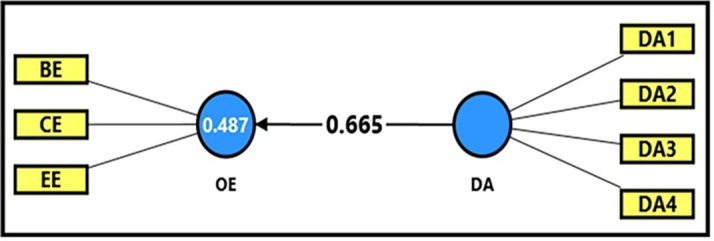
Analysis of the impact between the digital aesthetics dimension and productive organizational energy.

Table 13 and Figure 5 demonstrate the extracted (F) magnitude between digital aesthetics and productive organizational energy, which was recorded (273.197), greater than the tabular (F) magnitude of (3.94) at a significance level of (0.05), indicating a statistically significant impact of digital aesthetics on productive organizational energy. The findings indicate that digital aesthetics explain (48%) of the changes occurring in productive organizational energy, reflecting its role in stimulating positive interaction among employees through attractive and user-friendly digital interfaces. The extracted (t) magnitude for the digital aesthetics variable was also recorded (16.529), which was greater than the tabular magnitude (1.984) at a significance level of (0.05), indicating the significance of (β). This demonstrates that the magnitude (β = 0.665) indicates that an increase in digital aesthetics by one unit leads to a 67% increase in productive organizational energy by (67%), which confirms the importance of designing digital systems in a way that enhances interaction and motivation among employees in academic institutions.

*3.4.2.3 Third sub-hypothesis*

**Figure 6.**
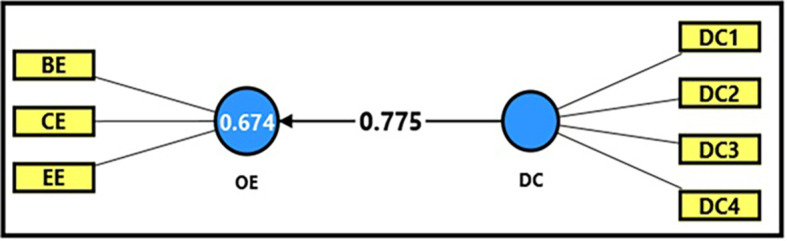
Analysis of the impact between the dimension of digital capabilities and productive organizational energy.

Table 13 and Figure 6 demonstrate the extracted (F) magnitude between digital capabilities and productive organizational energy, which recorded (594.552), greater than the tabular (F) magnitude of (3.94) at a significance level of (0.05), providing sufficient support to accept the alternative hypothesis stating a statistically significant impact of digital capabilities on productive organizational energy. It was observed that digital capabilities could explain (67%) of the changes occurring in productive organizational energy, indicating the role of employees’ technical capabilities in enhancing overall performance and achieving positive interaction within the organization. The extracted (t) magnitude for the digital capabilities’ variable was also recorded (24.383), which was greater than the tabular magnitude (1.984) at a significance level of (0.05), indicating the significance of (β). From the magnitude (β = 0.775), an increase in digital capabilities by one unit leads to an increase in productive organizational energy by (77%), which confirms that developing employees’ technical skills is a crucial factor in developing productive organizational energy and increasing work efficiency.

*3.4.2.4 Fourth sub-hypothesis*

**Figure 7.**
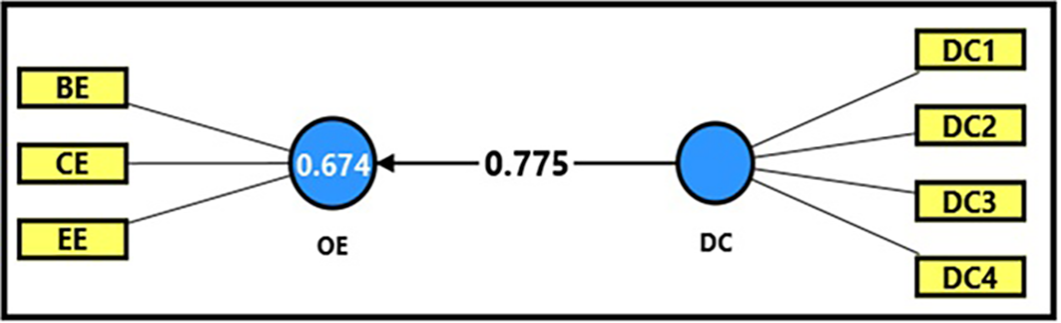
Analysis of the impact of digital intelligence dimension on productive organizational energy.

Table 13 and Figure 7 demonstrate the extracted (F) magnitude between digital intelligence and productive organizational energy, which was recorded (662.590), greater than the tabular (F) magnitude of (3.94) at a significance level of (0.05), which provides sufficient support for accepting the alternative hypothesis that digital intelligence has a statistically significant impact on productive organizational energy. The findings indicate that digital intelligence was able to explain 69% of the changes occurring in productive organizational energy, demonstrating its high capability to influence institutional performance levels through the effective utilization of smart technologies in analysis and decision-making. The extracted (t) magnitude for the digital intelligence variable was (25.741), which was greater than the table magnitude (1.984) at a significance level of (0.05), indicating the significance of (β). It is clear from the magnitude (β = 0.819) that an increase in digital intelligence by one unit leads to an 82% increase in productive organizational energy, which confirms that the employment of artificial intelligence and advanced digital analytics contributes to activating productive organizational energies and enhancing performance efficiency in academic institutions.

3.4.3 Second main hypothesis

**Figure 8.**
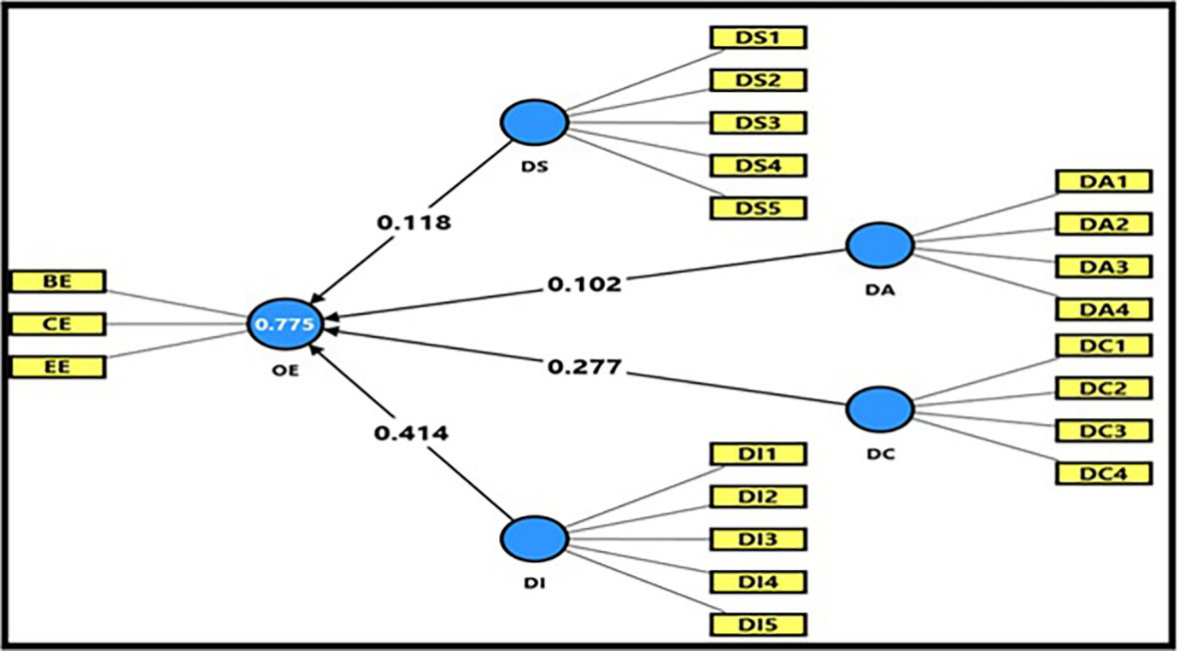
Impact analysis of digital workplace dimensions together on productive organizational energy.

Findings of Impact Analysis for Digital Workplace Dimensions and Productive Organizational Energy Table 14 and Figure 8 depict the detailed findings for impact analysis between Digital Workplace Dimensions and Productive Organizational Energy. The computed F magnitude (245.530) is higher than the table magnitude (2.46) at P = 0.05 level of significance. This means that the statistical model is statistically significant; further, the joint dimensions of the digital workplace help explain the variation in productive organizational energy. R-magnitude (0.880), which indicates a strong relationship between the variables used, and R ^2^-magnitude (0.775), which means that the dimensions of the digital workplace can explain (77.5%) of the differences in productive organizational energy. This is a reasonable proportion, indicating a high power to explain the model. The R ^2^ Adj magnitude was (0.772) indicating a good fit of the model and validating it for the analysis. As for the t-test, Table 8 indicates that all hypotheses were higher than the table magnitude (1.984) at the level of significance (0.05), indicating a statistically significant impact of most digital workplace dimensions to explain the variation occurring in PWE, yet with different levels of impact strength. Digital space had a t-magnitude of (2.581) at (β = 0.118), and the significance level was obtained as (Sig = 0.010), which means digital space moderately contributes to increases in productive organizational energy by promoting collaboration among employees and, hence, transfers frequent communication among employees. Digital aesthetics attained (t = 2.354) at (β = 0.102), and the level of significance was (Sig = 0.019), which is significant yet weak, suggesting that the aesthetic features of digital systems add somewhat to inspiring productive organizational energies by enhancing user experience and functional interaction only regarding limited weight for obtaining knowledge gains from a system design to produce work process innovations. On the other hand, the influence of digital capabilities was highly significant with a t-magnitude of (5.320) at (β = 0.277), and Sig-level 0.000, which confirms that employees’ ownership of technical capabilities as well as digital competencies contributes significantly to elevating the level of productive organizational energy and improving performance effectiveness. The highest effective dimension among the 4-dimensions was digital intelligence, as it acquired a t-magnitude = (8.332) at (β = 0.414) and significance magnitude = (Sig = 0.000), which means that the dependency of the university on analytic systems and artificial intelligence in conducting operations and coordinating information is the most useful element in triggering productive organizational energy, and maximizing the institutional performance effect. On the other hand, the findings of the multicollinearity test of (Tolerance 0.260 – 0.384 > 0.10) and (VIF 2.605 −3.852 <10) indicate that no problem of multicollinearity exists among all independent dimensions and indicates that the model is applicable for statistical analysis. Thus, overall, it can be concluded that the aggregate dimensions of the digital workplace do work for the development of productive organizational energy, but in varying degrees, ranging from strong impact being made by digital intelligence to, less impact being made by digital capabilities and space while limited influence was exerted by aesthetics. These findings explain that university institutions investing in smart technologies and improving employees’ ICT skills achieve better performance efficiency, stimulate the work environment, and facilitate cooperation and integration among new digital systems (intelligent networks), thereby receiving higher levels of productive organizational energy.

**Table 14.** Impact analysis of digital workplace dimensions together on productive organizational energy.

Dimensions of the digital workplace	α	β	t	Sig	R	R ^2^	(R ^2^) Adj	F	Sig	Tolerance	VIF
Digital Space	0.341	0.118	2.581	0.010	0.880	0.775	0.772	245.530	0.000	0.269	3.712
Digital Beauty		0.102	2.354	0.019						0.384	2.605
Digital Capabilities		0.277	5.320	0.000						0.260	3.852
Digital Intelligence		0.414	8.332	0.000						0.308	3.247

### 3.5 Heterotrait–Monotrait Ratio (HTMT) test – Digital workplace matrix

The Heterotrait–Monotrait ratio (HTMT) matrix showing the inter-trait correlation is provided in Table 15 for construct the digital workplace. The results demonstrate that all  HTMT values are less than the acceptable cut-off point (0.90), as suggested by ( Henseler et al., 2015), which  provides evidence of discriminant validity between the four dimensions of the digital workplace model. The coefficients are indicative of moderate relationships among dimensions, allowing for both conceptual and empirical separateness, but preserving logical unity within the construct. The HTMT analysis confirms that the digital workplace has good structural distinctiveness among its dimensions, which increases its conceptual robustness and justifies proceeding with SEM_ of the main study variables.

**Table 15.** HTMT test – Digital workplace.

	DA	DC	DI	DS
**DA**	—	—	—	—
**DC**	0.812	—	—	—
**DI**	0.748	0.826	—	—
**DS**	0.735	0.785	0.711	—

### 3.6 Heterotrait–Monotrait Ratio (HTMT) test – Organizational energy matrix

Table 16 presents the HTMT matrix of the dimensions of the organizational energy construct. This test serves the purpose of testing the discriminant validity between dimensions to ensure conceptual and empirical independence. The findings reveal that all HTMT values are smaller than the predefined 0.90 criterion ( Henseler et al., 2015), which suggests that the relationships between dimensions are moderate and acceptable, thereby demonstrating a high level of distinctiveness among these dimensions. These results confirm the theoretical coherence and structural validity of the model, demonstrating that, although the three dimensions are interrelated, they are statistically and conceptually different. The two models together provide a unified and linked partition of organizational energy within the context of university-related work.

**Table 16.** HTMT test – Organizational energy.

	DA	DC	DI	DS
DA	—	—	—	—
DC	0.812	—	—	—
DI	0.748	0.826	—	—
DS	0.725	0.815	0.831	—

## 4. Conclusions

The research offers strong empirical insight on the strategic significance of the digital workplace for productive organizational energy in higher education institutions, using the analytical case of University of Fallujah. The studies elucidated that the digital workplace of the university is located at an average-good level of development, indicating a growing tendency within the institution for digital transformation to increase performance and operational efficiency. In terms of dimensions, digital intelligence stands out as the leading advanced and influential factor, which means that knowledge about artificial intelligence applications and digital analytics to improve decision-making, productivity, and institutional outcomes is increasing. Digital literacies and digital aesthetics were at moderate levels, which confirms that there are satisfactory computing competencies of employees to work in organizations today while training plans accompanied by user friendly interfaces are still necessary for leveraging performance. Digital utilization level scored medium, suggesting that digital collaboration between different departments is still insufficient, and large channel-based workflow integration and interaction are required.

From an inferential standpoint, the regression analysis revealed a strong and significant influence of the digital workplace on productive organizational energy (β = 0.892, R ^2^ = 0.75, F = 865.962, p < 0.001), indicating that progression in the digital milieu significantly contributes to employees’ zeal, motivation, and engagement with the organization. Among the four dimensions of the digital workplace, digital intelligence had the maximum impact (β = 0.819, R ^2^ = 0.69), followed by digital capabilities (β = 0.775, R ^2^ = 0.67), digital space (β = 0.651, R ^2^ = 0.59), and digital aesthetics (β = 0.665, R ^2^ = 0.48). These results suggest that the digital workplace is not a location from which administrative tasks are supported; it is a place where emotional, cognitive, and behavioral forces converge to generate productive organizational energy. Additionally, the regression model exhibited high explanatory power and internal consistency, with no multicollinearity effect, confirming the robustness and reliability of the proposed theoretical framework.

## 5. Recommendations

1. It is necessary to enhance digital intelligence in the university work environment by investing in smart technologies and employing them in planning and decision-making.
2. Developing the digital capabilities of employees through continuous training and technical support to develop their skills in dealing with digital tools and platforms.
3. Improving the digital space environment by expanding the use of collaborative digital platforms and integrating administrative and academic units.
4. Attention should be paid to digital aesthetics by designing interactive and attractive digital interfaces that contribute to increasing job satisfaction and motivate employees to interact with electronic systems.
5. Implementing a holistic digital workplace model, considered through the four dimensions of the digital workspace with a single institutional vision to drive organizational performance and create an inspiring and efficient working environment.
6. The relative cultural context of Iraq, which is characterized by a hierarchical structure and collectiveness, moderates the effect of the digital workplace on organizational energy. Encouraging leadership cultivation, digital trust, and culturally embedded partnerships can foster employees emotional and cognitive engagement.
7. In data-poor environments, the use of open-source AI toolkits in combination with regional digital training and incremental capacity building facilitates optimal digital intelligence and sustainable institutional performance.

### Ethical considerations

This study involved human participants and was conducted in accordance with accepted ethical research standards and the principles outlined in the Declaration of Helsinki. Ethical approval was obtained from the Scientific Research Ethics Committee, University of Fallujah, Iraq (Approval No. HOF.HUM.2025.001). Written informed consent was obtained from all participants prior to their participation. All participants were informed about the purpose of the study, the voluntary nature of their participation, their right to withdraw at any time without consequences, and the confidentiality of their data.

## Informed consent

Written informed consent was obtained from all individual participants prior to their participation in the study. All participants were informed about the purpose of the research, their right to withdraw at any time, and the confidentiality of their data.

## Data Availability

The data supporting the findings of this study are openly available in Zenodo at: https://doi.org/10.5281/zenodo.18290375, Laith Emad, A., & Lateef Khalaf, Y. (2026). The Role of the Digital Workplace in Enhancing Productive Organizational Energy: An Analytical Study at the University of Fallujah. Zenodo. The data are available under the terms of the Creative Commons Attribution 4.0 International license (CC-BY 4.0). This study is observational survey-based research and follows the STROBE reporting guidelines. No CONSORT or ARRIVE checklists are required as the study does not involve clinical trials or animal experiments.
