# The Role of the Digital Workplace in Enhancing Productive Organizational Energy: An Analytical Study at the University of Fallujah

**DOI:** 10.12688/f1000research.174088.2

**Published:** 2026-05-19

**Authors:** Amna Laith Alhashimi, Yasser Lateef Khalaf

**Affiliations:** 1 Public Administration, University of Fallujah, Al-Fallujah, Al Anbar Governorate, Iraq

**Keywords:** Digital workplace, productive organizational energy, University of Fallujah.

## Abstract

**Background:**

Digital workplaces are increasingly adopted in higher education institutions as part of broader digital transformation initiatives. However, many universities still view digital workplace systems primarily as technological tools rather than integrated socio-organizational environments capable of supporting employee engagement and productive organizational energy. Despite growing interest in digital transformation, limited empirical research has examined how specific dimensions of the digital workplace influence organizational energy in higher education contexts. Therefore, the main aim of this paper is to provide comprehensive and sold empirical insight on the strategic significance of the digital workplace for productive organizational energy in higher education institutions, using an analytical case study of University of Fallujah.

**Methods:**

A descriptive-analytical research design were employed. Data were collected through a structured questionnaire from 290 respondents affiliated with the University of Fallujah. The data were analyzed using SPSS version 28 and Smart PLS 4. The statistical analysis included reliability testing, confirmatory factor analysis, and regression analysis to examine the relationships between digital workplace dimensions and productive organizational energy.

**Results:**

The findings indicate a strong positive association between the digital workplace and productive organizational energy (β = 0.892, R ^2^ = 0.75, p < 0.001). Among the examined dimensions, digital intelligence showed the strongest relationship with productive organizational energy (β = 0.819), followed by digital capabilities (β = 0.775), digital aesthetics (β = 0.665), and digital space (β = 0.651). These results suggest that advanced digital systems, employee digital competencies, and well-designed digital environments are closely associated with higher levels of emotional, cognitive, and behavioral energy among employees.

**Conclusions:**

This study contributes to the digital transformation by linking digital workplace dimensions with productive organizational energy in a higher education. The findings obtained in this paper highlight the importance of strengthening digital intelligence and enhancing employees’ digital capabilities to support employee engagement and institutional effectiveness in digitally transforming universities.

## 1. Introduction

The digital workplace concept has transformed immensely over the last 20 years, becoming a key cornerstone of business transformation. A digital Workplace is characterized as a combined set of devices, applications, and digital networks that allow employees to perform their tasks flexibly from anywhere bringing productivity and organizational agility ( de Moraes et al., 2024; Ujwary-Gil & Godlewska-Dzioboń, 2022). In the context of higher education institutions, digital transformation is a key factor for optimizing operations and communication, as well as service quality, and plays a critical role in both organizational innovation and user satisfaction ( Mishra et al., 2023; Too et al., 2025; Al-Rawi et al., 2021). Researchers have conceptualized the digital workplace as a vibrant ecology consisting of technological, social and organizational aspects that influence interaction and collaboration in media-saturated environments ( Chawla & Goyal, 2022; Marsh et al., 2024). The latest technological developments have changed the nature of traditional work designs, leading to enhanced remote collaboration, cross-functional integration, and employee performance and quality of life ( Meske & Junglas, 2021; Nesindande et al., 2024).

### 1.1 Digital Workplace Dimensions and Their Theoretical Relevance

The current literature indicates that the digital workplace enhances flexibility, connectivity, and inclusiveness through remote access, collaborative platforms, and evidence-based intelligence ( Attaran et al., 2019; Kaur et al., 2025; Rakovic et al., 2022). By doing so, such digital spaces advance employees’ engagement through collaboration and ongoing learning, comparing performance with one another. and provides analytical tools to managers ( Eckhardt et al., 2019; Hoert et al., 2018; Husien et al., 2018). Nevertheless, the application of digital workplaces is uneven throughout industries, especially in academia, where there is a tendency to characterize digitalization only as tool adoption and not from an organizational or behavioral perspective ( Zimmer et al., 2023). consequently, the potential benefits of digital technologies for organizational effectiveness and innovation are underexploited ( Khin & Ho, 2019; Usai et al., 2021).

To address this gap, this study performs a framework for digital workplace through four theoretically distinct dimensions, which is known as digital space, digital aesthetics, digital capabilities, and digital intelligence. Each one of these dimensions is argued to contribute uniquely to the activation of productive organizational energy. Understanding the individual theoretical contribution of each dimension to organizational outcomes is essential. Digital Space refers to the shared collaborative digital environment where employees can be interacted, communicated, and coordinated their work activities across administrative and academic units. Previous research works confirmed that digital spaces can be used to improve engagement through collaboration learning, while at the same time can provide managers with analytical tools to monitor and improve performance ( Eckhardt et al., 2019; Hoert et al., 2018). To this end, collaborative digital platforms facilitate cross-functional integration and reduce structural barriers between departments, which can directly improve employees’ enthusiasm, commitment, and sense of belonging within the organization ( Attaran et al., 2019; Rakovic et al., 2022). These findings align with the emotional dimensions of productive organizational energy. These indicated that such digital environments would create motivation and cooperative work behavior ( Cole et al., 2005; Vogel et al., 2022).

Digital Aesthetics on the other hand refers to the visual appeal, usability, and interactive quality of digital interfaces and systems through which employees engage with their work environment. Research on digital transformation indicates that the design of digital systems plays a meaningful role in shaping user experience and functional interaction, which in turn influences employees’ willingness to engage with and adopt digital tools ( Mishra et al., 2023; Chawla & Goyal, 2022). Attractive and user-friendly digital interfaces stimulate positive interaction among employees, reduce friction in daily work processes, and contribute to a motivating work atmosphere ( Marsh et al., 2024). These outcomes are theoretically linked to the emotional energy dimension of productive organizational energy, which encompasses enthusiasm, satisfaction, and the vitality employees bring to their work ( Baker, 2019; Gabardo-Martins, 2022). Therefore, one can concluded that digital aesthetics plays a significant positive impact on productive organizational energy.

Digital Capabilities denotes the technical expertise, digital proficiency, and skill levels that the employees can demonstrate in exploiting digital applications across their workplace. Previous studies shows that digital ability is a essential factor of organizational performance with employees’ technical skills operating as a bridging factor between digital technology adoption and organizational effectiveness ( Khin & Ho, 2019; Usai et al., 2021). Moreover, the skills enhancement of digital by means of ongoing training and technical assistance can improve employees’ intellectual willingness to transfer knowledge, proactively search for possibilities for self-development, and engage in creative problem-solving ( Hoert et al., 2018; Meske & Junglas, 2021). These findings align directly to the mental energy dimension of beneficial organizational energy, which incorporates knowledge-sharing and information exchange practices, intellectual engagement, and the potential for continuous attention and creative thinking aimed at organizational objectives ( Baker, 2019; Cole et al., 2005). The proposed digital capabilities demonstrate a considerable beneficial influence on productive organizational energy.

Digital Intelligence refers to the use of artificial intelligence tools by organization employs. It also can refer to the use of advanced analytics, smart technologies, and data-driven decision-making tools within its operational and administrative processes. Research studies on digital transformation highlights that the integration of intelligent technologies into organizational workflows can significantly enhance the decision-making, operational efficiency, and institutional responsiveness to change ( Khin & Ho, 2019; Zimmer et al., 2023). Furthermore, the availability of smart digital solutions is required to increase employee awareness and also to achieve performance improvement. It is also needed to support evidence-based planning, and allows more effective coordination for the organization ( Attaran et al., 2019; Chawla and Goyal, 2022). These findings are theoretically linked to the three dimensions of organizational productivity: affective, cognitive, and behavioral. Intelligent systems simultaneously enhance employee motivation, refine cognitive focus, and direct behavioral efforts toward clearly defined organizational goals ( Baker, 2019; Alexiou et al., 2019; Vogel et al., 2022). Therefore, it is assumed that digital intelligence has the strongest and most significant positive impact on organizational productivity among all four dimensions of the digital work environment.

### 1.2 Productive Organizational Energy and Its Theoretical Foundation

The notion of productive organizational energy has been identified as a crucial aspect of institutional vitality and effectiveness ( Abualhamael, 2017; Alexiou et al., 2019; Cole et al., 2005; Obeng et al., 2021; Husien et al., 2019). This refers to the set of emotional, cognitive, and behavioral resources through which creativity, focus, and resilience contribute to achieving organizational objectives ( Baker, 2019; Gabardo-Martins, 2022). Prior research indicates that high levels of OE strengthen job satisfaction, commitment, and collaborative performance, thereby fostering institutional competitive advantage ( Cuff & Barkhuizen, 2014; Vogel et al., 2022). Notwithstanding these insights, there is limited_ empirical evidence associating the dimensions of the digital workplace—namely, digital space, capabilities, aesthetics, and intelligence – to the activation of productive organizational energy, especially in higher education institutions in developing countries ( Kelder et al., 2025; White, 2012; Al-Sabaawe et al., 2020).

### 1.3 Linking Digital Workplace to Productive Organizational Energy: The Research Gap

Empirical evidence is limited, which links the dimensions of the digital work environment digital space, capabilities, aesthetics, and intelligence to the activation of productive organizational power, particularly in higher education institutions in developing countries ( Kelder et al., 2025; White, 2012; Al-Sabaawe et al., 2020). While previous studies have addressed the impact of digital transformation on organizational performance in general ( Khin and Ho, 2019; Usai et al., 2021), and others have explored organizational power in the context of leadership and job satisfaction ( Abualhamael, 2017; Obeng et al., 2021), no study has yet explicitly addressed the four dimensions of the digital work environment as distinct predictors of the three components of productive organizational power affective, cognitive, and behavioral within a higher education institution in the context of a developing Arab country.

This research bridges _this gap and explores how digital workplaces influence the productive organizational energy of the University of Fallujah. This study seeks to broaden the theoretical perspectives of digital workplaces by intertwining them with the theory of the organizational energy field, to understand how digital transformation impacts employees’ emotional, cognitive, and behavioral engagement. The empirical research in this paper has both theoretical and application dimensions for the evolution of digital transformation literature as well as practice for institutions of higher education regarding how they could best design their digital space to achieve motivation, collaboration, and overall organizational performance.

Building on the theoretical foundations outlined above, this study proposes that the four dimensions of the digital workplace, digital space, digital aesthetics, digital capabilities, and digital intelligence, each exert a statistically significant and positive impact on productive organizational energy at the University of Fallujah. Specifically, it is hypothesized that digital space, as a shared collaborative digital environment, enhances employees’ enthusiasm and commitment levels, thereby activating productive organizational energy. Similarly, digital aesthetics, through attractive and user-friendly digital interfaces, is hypothesized to stimulate positive interaction and motivation among employees. Digital capabilities, reflecting employees’ technical proficiency and digital competencies, are hypothesized to contribute significantly to elevating productive organizational energy and improving performance effectiveness. Furthermore, digital intelligence, through the effective employment of artificial intelligence and advanced digital analytics in analysis and decision-making, is hypothesized to represent the most influential dimension in activating productive organizational energies and enhancing institutional performance efficiency. In addition to these sub-hypotheses, an overarching main hypothesis proposes that the digital workplace as a whole encompassing all four dimensions simultaneously, exerts a strong and significant collective impact on productive organizational energy, whereby emotional, cognitive, and behavioral forces converge within the digital work environment to generate institutional vitality and effectiveness.

## 2. Methods

Research Methodology: The researchers in their study relied on a descriptive analytical method, which is considered appropriate for providing a clear and accurate picture of the phenomenon of the research problem. This is because it studies the characteristics and forms of events and phenomena and combines more than one approach at the same time, represented by observations, questionnaires, and personal interviews, which directly access information.

The descriptive-analytical method is selected in this study because it is considered appropriate for providing a clear and accurate vision of a research, as it studies the characteristics and forms of events and combines multiple approaches simultaneously, including observations, questionnaires, and personal interviews, which allow direct access to information ( Mishra et al., 2023; Marsh et al., 2024). This approach is widely recognized in organizational and digital transformation studies as suitable for examining relationships between variables within a specific institutional context, particularly when the aim is to measure perceptions and behaviors of employees within a university setting ( Meske & Junglas, 2021; Kelder et al., 2025). Furthermore, the study employed SPSS v.28 and Smart PLS v.4 for data analysis, utilizing confirmatory factor analysis and multiple regression, which are well-established analytical techniques in behavioral and organizational research ( Henseler et al., 2015).

Data were gathered using a structured questionnaire developed and adapted from established scales in the existing literature on digital workplaces and productive organizational energy. The digital workplace variable was measured across four dimensions: digital space, digital aesthetics, digital capabilities, and digital intelligence, comprising 18 items in total, which were adapted from prior studies on digital workplace design and transformation ( Marsh et al., 2024; Attaran et al., 2019; Meske & Junglas, 2021). The productive organizational energy variable was measured across three dimensions: emotional energy, cognitive energy, and behavioral energy, comprising 17 items in total, adapted from validated organizational energy scales in the literature ( Cole et al., 2005; Gabardo-Martins, 2022; Vogel et al., 2022). All items were rated on a five-point Likert scale to capture the degree of agreement or perception of respondents regarding each statement. The content validity of the instrument was ensured through alignment with the theoretical constructs defined in the literature, while construct reliability was confirmed through Cronbach’s alpha coefficients, which exceeded the acceptable threshold of 0.70 across all dimensions of both variables. Composite reliability (CR) values equally met acceptable standards, ranging between 0.874 and 0.924 for the digital workplace variable and between 0.899 and 0.937 for the productive organizational energy variable. Average variance extracted (AVE) values for all dimensions exceeded the standard threshold of 0.50, further confirming convergent validity of the instrument. Discriminant validity was additionally confirmed through the Heterotrait-Monotrait Ratio (HTMT) test, where all values remained below the 0.90 cut-off criterion recommended by Henseler et al. (2015). The results indicate statistical associations between variables rather than definitive causal relationships since the data were collected using a cross-sectional survey design.

### 2.1 Sample characterization

**2.1.1 Gender**

Table 1 displays the frequency distribution of the sample members by gender at the University of Fallujah. There were 290 sample members, and most of the sample (80.3%) was male (233 members). Only 57 participants were female (19.7%). Female involvement in the study was much lower than male participation, which may be due to the university’s largely male functional or academic organization.

**Table 1.** Frequency distribution by gender.

Gender	Frequency	Percentage (%)
Male	233	80.3
Female	57	19.7
Total	290	100.0

**2.1.2 Age**

Table 2 shows the frequency distribution of the sample members according to the age group at the University of Fallujah.

**Table 2.** The distribution by age.

Age group	Frequency	Percentage (%)
25-30	55	19.0
31-35	45	15.5
36-40	46	15.9
41-45	45	15.5
46-50	43	14.8
51-55	34	11.7
Higher than 55	22	7.6
Total	290	100

**2.1.3 Academic achievement**

Table 3 shows the frequency distribution of the sample members according to their academic achievements at the University of Fallujah.

**Table 3.** The distribution based on level of academic achievement.

Academic achievement	Frequency	Percentage (%)
Bachelor's Degree	87	30.0
Higher Diploma	48	16.6
Master's Degree	77	26.6
Doctorate	78	26.9
Total	290	100

**2.1.4 Years of service**

Table 4 presents the frequency distribution of the respondents according to their years of service at the University of Fallujah.

**Table 4.** The frequencies by years of service.

Years of service	Frequency	Percentage (%)
Less than 5 years	55	19.0
6–10 years	98	33.8
11–15 years	34	11.7
16–20 years	82	28.3
More than 20 years	21	7.2
**Total**	**290**	**100**

**2.1.5 Administrative position**

Table 5 shows the frequency distribution of the sample members according to their administrative positions at the University of Fallujah. The research sample was a balanced representation of all administrative and academic levels, with a clear dominance of teaching staff. This lends the study’s findings to academic depth and provides a realistic reflection of the university’s organizational structure.

**Table 5.** The frequency distribution by administrative position.

Administrative position	Frequency	Percentage (%)
Employee	45	15.5
Faculty Member	105	36.2
Division Head	56	19.3
Department Head	76	26.2
Dean	4	1.4
Assistant Dean	4	1.4
**Total**	**290**	**100**

**2.1.6 Academic rank**

Table 6 presents the frequency distribution of the respondents according to their academic rank at the University of Fallujah. The sample tends towards higher academic levels, as the categories (Professor, Assistant Professor, and Assistant Lecturer) constitute more than two-thirds of the sample, which gives the study results cognitive depth and scientific rigor stemming from advanced academic experiences within the university.

**Table 6.** The frequency distribution by academic rank.

Academic rank	Frequency	Percentage (%)
None	46	15.9
Professor	78	26.9
Associate Professor	68	23.4
Lecturer	40	13.8
Assistant Lecturer	58	20.0
**Total**	**290**	**100**

## 3. Results and discussion

### 3.1 Response rate

To ensure fulfillment of the study’s requirements, the researcher distributed (300) questionnaires to a random sample of affiliates at Fallujah University. 290 were retrieved and validated for statistical analysis, as shown in Table 7.

**Table 7.** The number of questionnaires distributed.

Status	Number of distributed questionnaires	Number of unreturned questionnaires	Number of valid questionnaires for analysis
Number	300	10	290
Percentage	100	3.333	96.67

### 3.2 Results of the descriptive analysis

The conclusions regarding the responses of the sample were based on reviewing the information and analyzing it using statistical methods. The findings will be discussed in light of the current research axes, and an effort will be made to interpret these relationships and findings.

**3.2.1 Digital workplace independent variable**

According to Table 8, the digital workplace variable featured relative importance order (first), overall arithmetic mean (3.350), standard deviation (PCS = 0.805), coefficient of variation (24.04%), and medium evaluation level. This therefore indicates that the productive organizational ‘energy’ environment is keen to embrace digital workplace technologies ( Marsh et al., 2024). However, this level is not of strong adoption, but moderate inclination towards digital transformation, which may be due to technical or organizational barriers such as limited technical resources and institutional unawareness ( Mishra et al., 2023). The highest mean of (3.424), SD = 0.845, and CV = (24.67%) were reported for digital intelligence and attained a level classification of (good). This indicates an interest in using intelligent technologies to analyze data and make decisions, which can be considered as a progress of awareness within the university about the role of smart digital solutions, although it is not experienced at the same level by all administrative units. Second, digital aesthetics had a median score of 3.386, standard deviation of 0.870, and coefficient of variation of 25.70%, all at (medium) level of rating. This indicates a kind of tension of the attractive power of digital systems to look, as well as being interacted with by users. These efforts, however, need to be better infused into the interface design and user experience for a more meaningful impact on increasing work efficiency. Meanwhile, the digital (3.294), std. Focused capabilities were third with 0.878 std and a variation coefficient of (26.65%) therefore, an evaluation tier of (Medium) levels despite over the actionable level these capabilities should be posed in evaluating as they have, on average, some high impact and important (Medium) level, but also with possible higher potential development. This represents a modest level of digital proficiency among employees ( Hoert et al., 2018; Meske & Junglas, 2021). However, it also means that it is important to extend the training and skills programmed to improve the technical efficiency necessary to handle digitalization. Regarding digital, it obtained the lowest (average = 3.297, SD = 0.983, and CoV = 29.80%), with a rating level of (medium). This could mean that digital shared workspaces are not yet widespread and/or that there might be some limitations in terms of infrastructure or the availability of fully integrated digital systems. From this analysis, we can conclude that the measurement findings confirm the digital workplace variable at a medium scale biased towards positivity, with a higher advantage of pillar maturity appearing in digital intelligence rather than the others, as well as benchmarking scale by taking more development on training, interaction design, and digital infrastructure to establish an efficient and sustainable environment for work ( Marsh et al., 2024).

**Table 8.** Descriptive statistics for research variables and dimensions.

Research variables and dimensions	M	S	CV	Rank	Answer trend
Digital Space	3.297	0.983	29.80	4	Average
Digital Beauty	3.386	0.870	25.70	2	Average
Digital Capabilities	3.294	0.878	26.65	3	Average
Digital Intelligence	3.424	0.845	24.67	1	Good
Digital Workplace	3.350	0.805	24.04	First	Average
Emotional Energy	3.487	0.769	22.04	1	Good
Cognitive Energy	3.376	0.921	27.28	2	Average
Behavioral Energy	3.349	0.989	29.54	3	Average
Organizational Energy	3.404	0.829	24.35	Second	Average

1. Productive Organizational Energy Dependent Variable

Findings ( Table 8) revealed that the productive organization energy variable came at the second order regarding relative importance with a total arithmetic mean and a standard deviation of (3.404), and (0.829), respectively, equal to (.3435), a medium evaluation level. This implies a positive interest in the utilization of effective organizational energy practices within the work organization of universities. However, this level does not indicate high organizational vitality, but simply a situation of lack of negative as well as the presence of a low amount of positive interaction that can be enhanced by creating a motivating work environment and concentration on both employees’ behavioral and emotional energy sources ( Hoert et al., 2018; Meske & Junglas, 2021). Based on the analysis of dimensions, emotional energy held first place with an average (3.487), standard deviation (0.769) and, coefficient of variation (22.04%), and evaluated level. This is evidence of the good state of enthusiasm and satisfaction enjoyed by staff members, while working with vitality and activity, which means the availability of an inspiring campus atmosphere that reflects a spirit of pride and sense of belonging to the university. Cognitive energy arrived in second with an arithmetic mean of (3.376), a standard deviation of (0.921), and a coefficient of variation of (27.28%) along with a medium evaluation level. This is indicative of relatively high cognitive readiness among employees to share knowledge and actively look for opportunities to continuously develop themselves ( Hoert et al., 2018). However, the high coefficient of variation means that there is still a disproportion in this area among individuals, so we must reinforce the culture of organizational learning and intellectual participation. On the other hand, behavioral energy ranked third with (3.349) as an average difference, (0.989) as a standard deviation, and (29.54%) coefficient of variation for the previous evaluation level attempting to measure this variable means that the nature of its application in practical work still needs to be improved, where average measures indicate that this level lacks initiative or practical activity, especially since there is a discrepancy in efforts between employees. To instill a spirit of dedication and willpower, it calls for incessant encouragement, guidance, and organizational support. The analysis findings suggest that the organizational productive energy variable is positioned at an intermediate level of intensity, based on emotional energy, which translates into the main motivating source for employees to outperform, with cognitive and behavioral energies requiring more long-term development programs of motivation-enhancement in support of new forms of organizational learning and effective behavior within the university work context ( Hoert et al., 2018).

### 3.3 Confirmatory construct validity

**3.3.1 Confirmatory factor analysis for digital workplace**

**Figure 1.**
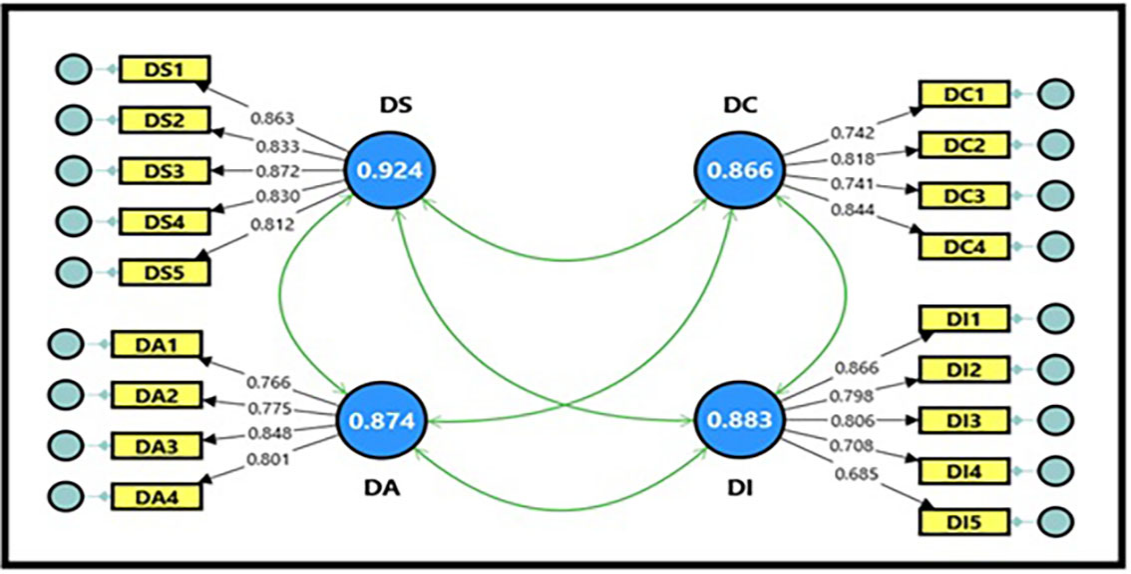
Confirmatory factor analysis of digital workplace.

As an independent variable, the digital workplace model consisted of four main dimensions: (digital space, digital aesthetics, digital capabilities, and digital intelligence) with 18 questions, as demonstrated in Figure 1. Table 9 shows the extracted fit quality indicators, which were within the required standards for model acceptance. All composite reliability (CR) magnitudes for the digital workplace variable were within acceptable limits, reaching (0.874, 0.867, 0.886, and 0.924), which is a good indicator and denotes the reliability of the scale, reflecting a high internal consistency of the scale’s dimensions ( Marsh et al., 2024). It is also clear from the Cronbach’s alpha coefficient magnitudes, which reached (0.874, 0.866, 0.883, and 0.924), that they are greater than (0.70), indicating high reliability between items for each dimension of the model. The findings demonstrated that all average variance extracted (AVE) magnitudes for the digital workplace variable were acceptable, ranging between (0.637, 0.621, 0.602, and 0.710), which is greater than the standard magnitude (0.50), indicating that the sub-dimensions contribute significantly to explaining the total variance of the digital workplace variable; thus, the model is considered more reliable in interpreting the relationships between its dimensions.

**Table 9.** Fit quality indicators for digital workplace.

Dimensions	Cronbach’s alpha (standardized)	Cronbach’s alpha (unstandardized)	Composite reliability (rho_c)	Average variance extracted (AVE)
DA	0.874	0.873	0.874	0.637
DC	0.866	0.865	0.867	0.621
DI	0.883	0.883	0.886	0.602
DS	0.924	0.924	0.924	0.710

Table 10 shows the estimation magnitudes, which ranged between (0.685 and 0.872) for all items representing the dimensions of the digital workplace variable. All items have high factor loading coefficients and exceed the acceptable minimum limit of (0.50), indicating that all items have a clear impact on their dimensions. It is also evident from the T-statistic magnitudes, which ranged between (12.288 and 19.761), that all of them are greater than the tabular magnitude of (1.984) at a significance level of (0.05), which is a sufficient indicator for adopting the model in its final form for subsequent analyses, and reflects the strength of the relationships between the items and the constituent dimensions of the digital workplace variable ( Marsh et al., 2024).

**Table 10.** Estimates for the dimensions of the digital workplace variable.

Paragraphs	Parameter estimates	Standard errors	T magnitudes	P magnitudes
DA1 <- DA	0.766	n/a	n/a	n/a
DA2 <- DA	0.775	0.082	13.514	0.000
DA3 <- DA	0.848	0.076	14.399	0.000
DA4 <- DA	0.801	0.072	14.044	0.000
DC1 <- DC	0.742	n/a	n/a	n/a
DC2 <- DC	0.818	0.084	14.031	0.000
DC3 <- DC	0.741	0.086	12.288	0.000
DC4 <- DC	0.844	0.078	14.277	0.000
DI1 <- DI	0.866	n/a	n/a	n/a
DI2 <- DI	0.798	0.053	17.029	0.000
DI3 <- DI	0.806	0.051	16.660	0.000
DI4 <- DI	0.708	0.051	13.568	0.000
DI5 <- DI	0.685	0.053	13.035	0.000
DS1 <- DS	0.863	n/a	n/a	n/a
DS2 <- DS	0.833	0.051	18.679	0.000
DS3 <- DS	0.872	0.052	19.761	0.000
DS4 <- DS	0.830	0.055	17.876	0.000
DS5 <- DS	0.812	0.055	17.397	0.000

**3.3.2 Confirmatory factor analysis of productive organizational energy**

**Figure 2.**
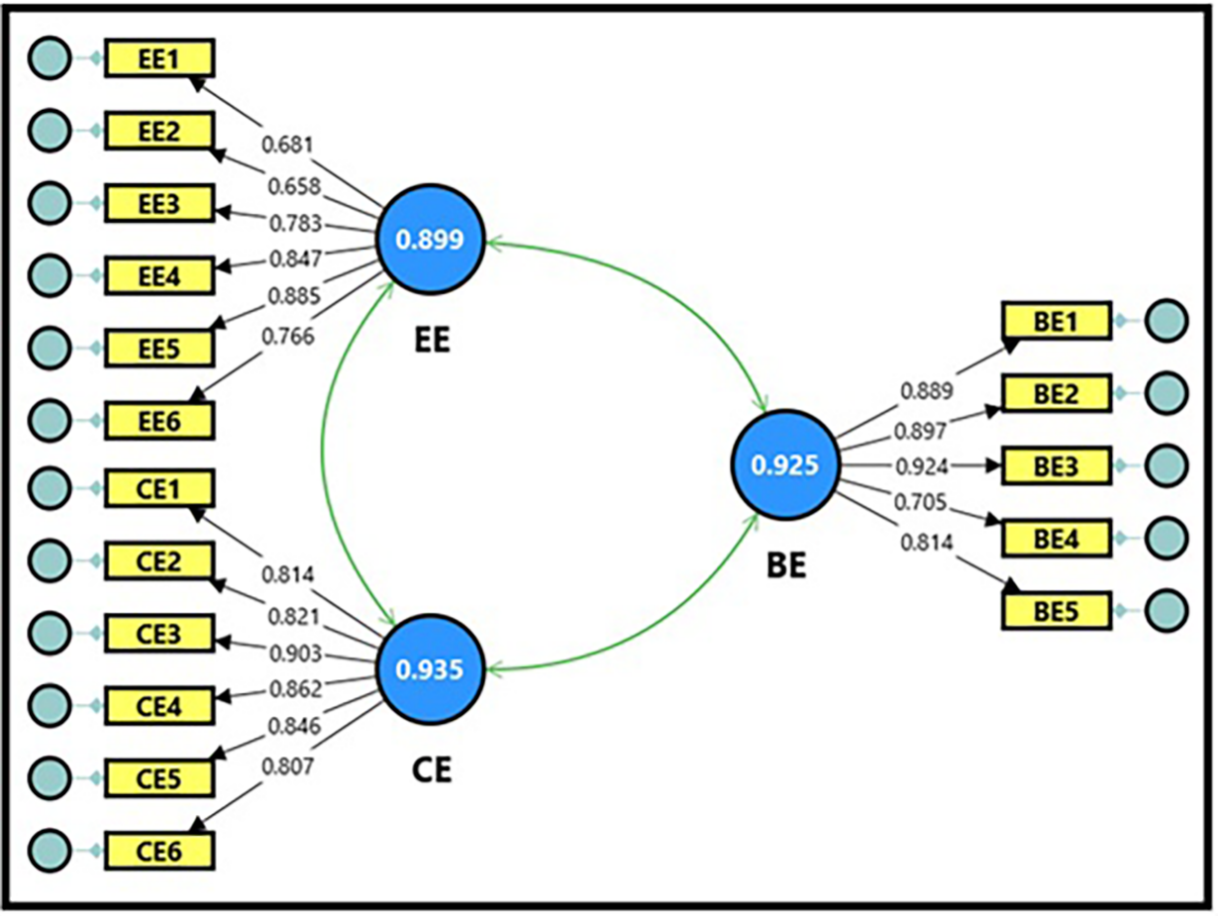
Confirmatory factor analysis of productive organizational energy.

The productive organizational energy model, as a variable, consists of three basic dimensions: behavioral, cognitive, and emotional energy, with (17) questions, as demonstrated in Figure 2. Table 11 shows the extracted fit quality indicators, which were within the required standards for model acceptance. The composite reliability (CR) magnitudes for the productive organizational energy variable were all within acceptable limits, reaching (0.929, 0.937, and 0.899), which is a good indicator and denotes the reliability of the scale, reflecting a high internal consistency of the scale’s dimensions. It is also clear from the Cronbach’s alpha magnitudes, which were (0.925, 0.935, and 0.899), which were greater than (0.70), indicating high reliability between the items for each dimension of the model. The findings demonstrated that the average variance extracted (AVE) magnitudes for the productive organizational energy variable were all acceptable, ranging between (0.599 and 0.722), which is greater than the standard magnitude (0.50), indicating that the sub-dimensions contribute significantly to explaining the total variance of the productive organizational energy variable; thus, the model is considered more reliable for interpreting the relationships between its dimensions.

**Table 11.** Fit quality indicators for productive organizational energy.

Dimensions	Cronbach’s alpha (standardized)	Cronbach’s alpha (unstandardized)	Composite reliability (rho_c)	Average variance extracted (AVE)
BE	0.925	0.925	0.929	0.722
CE	0.935	0.935	0.937	0.711
EE	0.899	0.898	0.899	0.599

Table 12 demonstrates the factor loading magnitudes, which ranged between (0.658 and 0.924) for all items comprising the dimensions of the productive organizational energy variable. All items were influential and had high factor loading coefficients exceeding the minimum acceptable limit of (0.50), reflecting the quality of their representation of their dimensions. It is also clear from the T-statistic magnitudes, which ranged between (10.592 and 24.110), that they were all greater than the tabular magnitude of (1.984) at a significance level of (0.05), which is considered a sufficient indicator for adopting the model in its final form in subsequent analyses, and reflects the strength of the correlation between the items and the dimensions comprising the productive organizational energy variable.

**Table 12.** Estimates for the dimensions of the productive organizational energy variable.

Items	Parameter estimates	T magnitudes	P magnitudes
BE1 <- BE	0.889	n/a	n/a
BE2 <- BE	0.897	22.742	0.000
BE3 <- BE	0.924	24.110	0.000
BE4 <- BE	0.705	14.511	0.000
BE5 <- BE	0.814	18.605	0.000
CE1 <- CE	0.814	n/a	n/a
CE2 <- CE	0.821	16.517	0.000
CE3 <- CE	0.903	18.980	0.000
CE4 <- CE	0.862	17.677	0.000
CE5 <- CE	0.846	17.356	0.000
CE6 <- CE	0.807	16.041	0.000
EE1 <- EE	0.681	n/a	n/a
EE2 <- EE	0.658	10.592	0.000
EE3 <- EE	0.783	12.307	0.000
EE4 <- EE	0.847	12.976	0.000
EE5 <- EE	0.885	13.338	0.000
EE6 <- EE	0.766	11.752	0.000

### 3.4. Testing research hypotheses

Testing the hypothesis between the dimensions of the (digital workplace) variable in the (productive organizational energy) variable is, shown in Table 13.

**Table 13.** Analysis of the impact between digital workplace dimensions on productive organizational energy.

Dependent variable	Sig	F	R ^2^Adj	(R ^2^)	R	t	Dimensions of the digital workplace		
**Organizational Productive Energy**	**0.000**	422.364	0.593	0.595	0.771	11.563	1.259	**α**	Digital Space
						20.552	0.651	**β**	
	**0.000**	273.197	0.485	0.487	0.698	8.205	1.153	**α**	Digital Beauty
						16.529	0.665	**β**	
	**0.000**	594.552	0.673	0.674	0.821	7.847	0.85	**α**	Digital Capabilities
						24.383	0.775	**β**	
	**0.000**	662.59	0.696	0.697	0.835	5.336	0.599	**α**	Digital Intelligence
						25.741	0.819	**β**	
	**0.000**	865.962	0.75	0.75	0.866	3.989	0.416	**α**	Digital Workplace
						29.427	0.892	**β**	

**3.4.1 The first main hypothesis**

**Figure 3.**
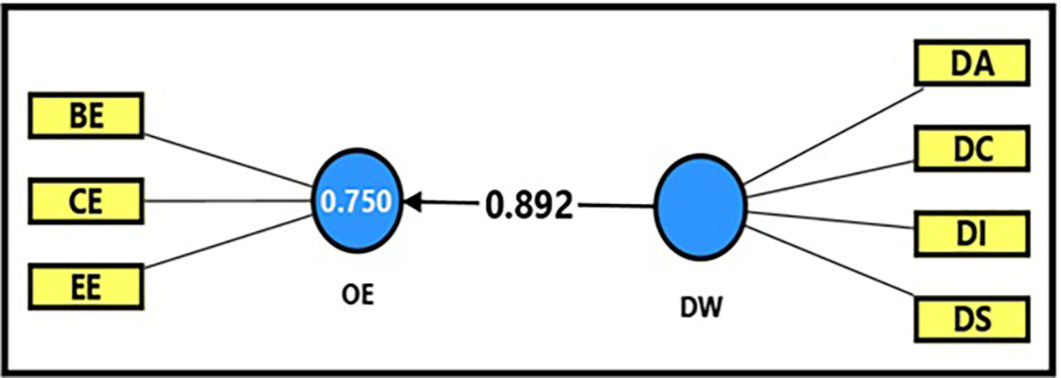
Analysis of the impact of the digital workplace on productive organizational energy.

It is clear from Table 13 and Figure 3 that the extracted (F) magnitude between the digital workplace and productive organizational energy was (865.962), which is greater than the tabular (F) magnitude of (3.94) at a significance level of (0.05). This result provides sufficient support for accepting the alternative hypothesis, which states that the digital workplace has a statistically significant impact on productive organizational energy. This indicates that the digital workplace has a strong significant impact on enhancing productive organizational energy, as it was able to explain (75%) of the changes occurring in productive organizational energy. In addition, the extracted (t) magnitude for the digital workplace variable was (29.427), which was greater than the tabular magnitude (1.984) at a significance level of (0.05), indicating the significance of (β) for the digital workplace variable. Noting that a one-unit enhancement in the digital workplace can be related to increase of 89% in productive organizational energy (β = 0.892). This would reflect a strong positive relation between the variables in the case stay used in this research, which is denoted by of the University of Fallujah. Besides, the digital work environment can be considered as a one of the most dominated factors that associated with the activation of productive organizational energy and performance improvement of institution. This finding deserves further investigation through coming research to establish the direction and stability of these relationships over time.

**3.4.2 Testing sub-hypotheses for digital workplace dimensions in productive organizational energy**

***3.4.2.1 First sub-hypothesis***

**Figure 4.**
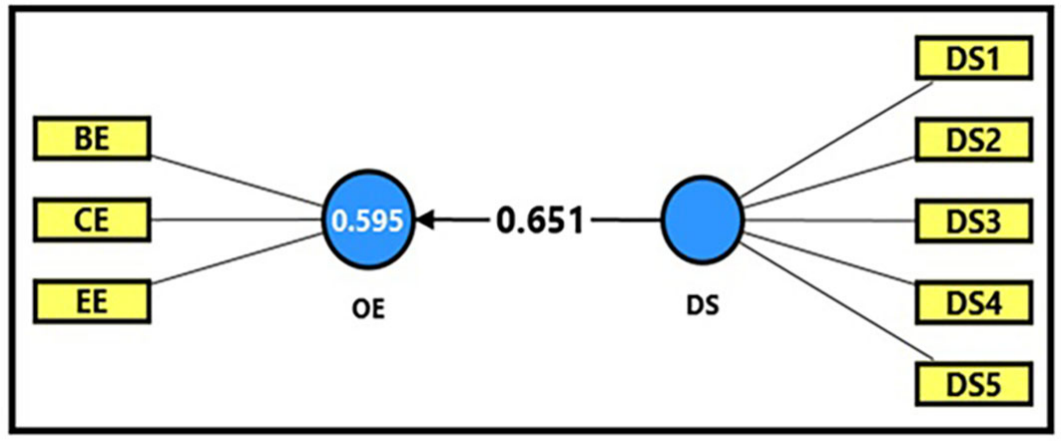
Analysis of the impact between the digital space dimension and productive organizational energy.

Table 13 and Figure 4 demonstrate the extracted (F) magnitude between digital space and productive organizational energy, which recorded (422.364), greater than the tabular (F) magnitude of (3.94) at a significance level of (0.05). This result provides sufficient support for the alternative hypothesis that digital space has a statistically significant impact on productive organizational energy. This indicates that digital space has a clear and significant impact on activating productive organizational energy, as it was able to explain (59%) of the changes occurring in it. The extracted (t) magnitude for the digital space variable was also recorded (20.552), which was greater than the tabular magnitude (1.984) at a significance level of (0.05), indicating the significance of (β) for the digital space variable. Noting that a one-unit enhancement in the digital workplace can be related to increase of 65% in productive organizational energy (β = 0.651). This would reflect a strong positive of shared digital work environments in relation to enthusiasm and commitment levels within academic institutions, which devoted here by the case stay used in this research, which is denoted by of the University of Fallujah.

***3.4.2.2 Second sub-hypothesis***

**Figure 5.**
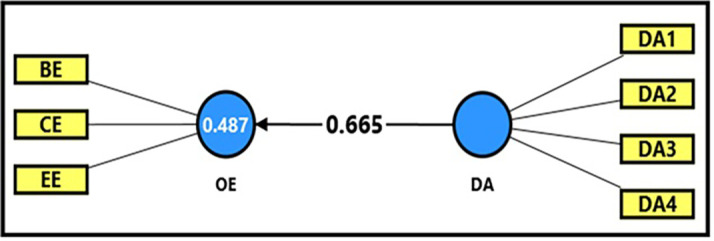
Analysis of the impact between the digital aesthetics dimension and productive organizational energy.

Table 13 and Figure 5 demonstrate the extracted (F) magnitude between digital aesthetics and productive organizational energy, which was recorded (273.197), greater than the tabular (F) magnitude of (3.94) at a significance level of (0.05), indicating a statistically significant impact of digital aesthetics on productive organizational energy. The findings indicate that digital aesthetics explain (48%) of the changes occurring in productive organizational energy, reflecting its role in stimulating positive interaction among employees through attractive and user-friendly digital interfaces. The extracted (t) magnitude for the digital aesthetics variable was also recorded (16.529), which was greater than the tabular magnitude (1.984) at a significance level of (0.05), indicating the significance of (β). Noting that a one-unit enhancement in the digital workplace can be related to increase of 67% in productive organizational energy (β = 0.665). This would reflect a strong positive relation of design quality of digital systems to interaction and motivation levels among employees in academic institutions, which devoted here by the case stay used in this research, which is denoted by of the University of Fallujah.

***3.4.2.3 Third sub-hypothesis***

**Figure 6.**
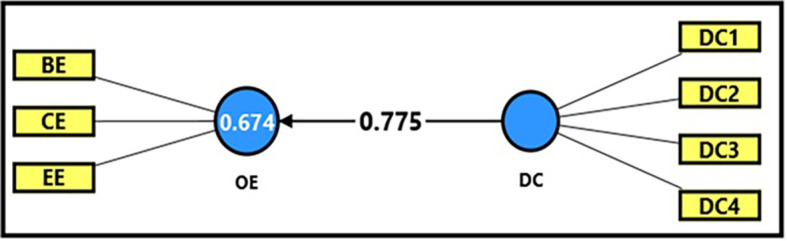
Analysis of the impact between the dimension of digital capabilities and productive organizational energy.

Table 13 and Figure 6 demonstrate the extracted (F) magnitude between digital capabilities and productive organizational energy, which recorded (594.552), greater than the tabular (F) magnitude of (3.94) at a significance level of (0.05), providing sufficient support to accept the alternative hypothesis stating a statistically significant impact of digital capabilities on productive organizational energy. It was observed that digital capabilities could explain (67%) of the changes occurring in productive organizational energy, indicating the role of employees’ technical capabilities in enhancing overall performance and achieving positive interaction within the organization. The extracted (t) magnitude for the digital capabilities’ variable was also recorded (24.383), which was greater than the tabular magnitude (1.984) at a significance level of (0.05), indicating the significance of (β). Noting that a one-unit enhancement in the digital workplace can be related to increase of 77% in productive organizational energy (β = 0.775). This would reflect a strong positive relation of employees’ technical skills and digital competencies to productive organizational energy and work efficiency which devoted here by the case stay used in this research, which is denoted by of the University of Fallujah.

***3.4.2.4 Fourth sub-hypothesis***

**Figure 7.**
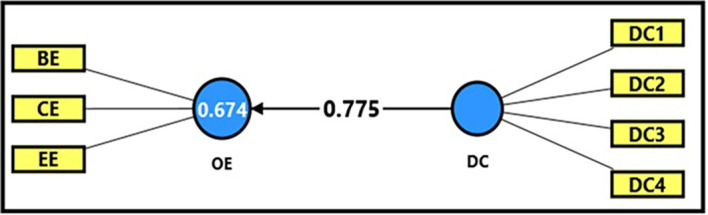
Analysis of the impact of digital intelligence dimension on productive organizational energy.

Table 13 and Figure 7 demonstrate the extracted (F) magnitude between digital intelligence and productive organizational energy, which was recorded (662.590), greater than the tabular (F) magnitude of (3.94) at a significance level of (0.05), which provides sufficient support for accepting the alternative hypothesis that digital intelligence has a statistically significant impact on productive organizational energy. The findings indicate that digital intelligence was able to explain 69% of the changes occurring in productive organizational energy, demonstrating its high capability to influence institutional performance levels through the effective utilization of smart technologies in analysis and decision-making. The extracted (t) magnitude for the digital intelligence variable was (25.741), which was greater than the table magnitude (1.984) at a significance level of (0.05), indicating the significance of (β). Noting that a one-unit enhancement in the digital workplace can be related to increase of 82% increase in productive organizational energy (β = 0.819). This would reflect a strong positive relation of the use of artificial intelligence and advanced digital analytics to productive organizational energies and performance efficiency in academic institutions, which is denoted by of the University of Fallujah.

**3.4.3 Second main hypothesis**

**Figure 8.**
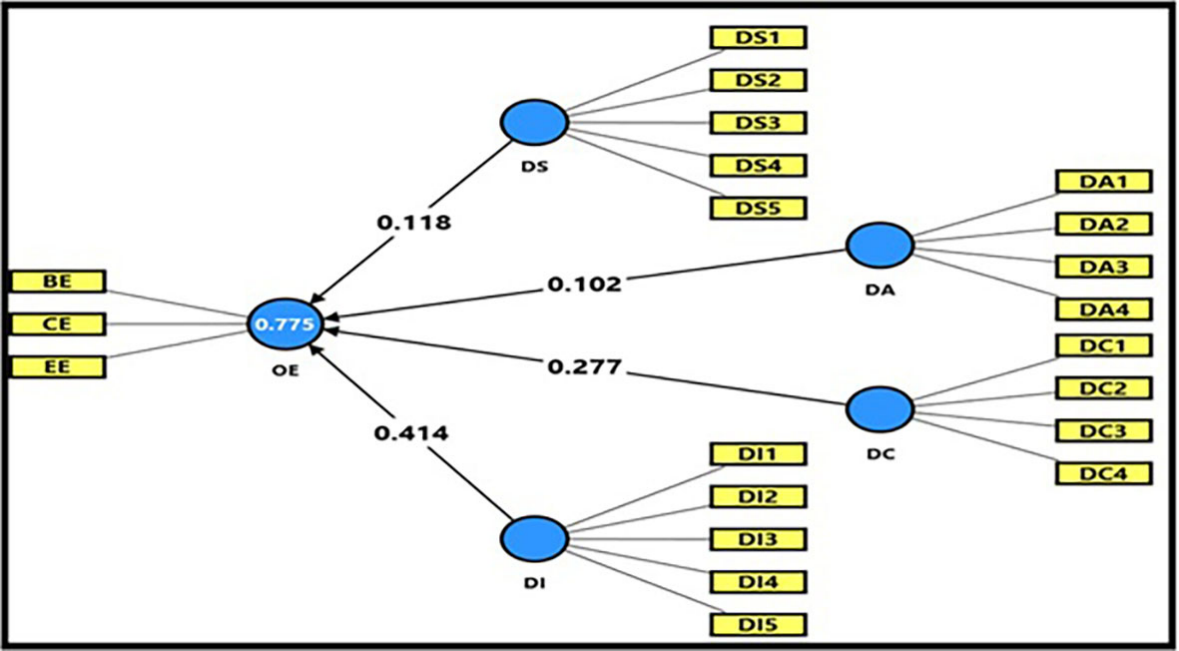
Impact analysis of digital workplace dimensions together on productive organizational energy.

Findings of Impact Analysis for Digital Workplace Dimensions and Productive Organizational Energy Table 14 and Figure 8 depict the detailed findings for impact analysis between Digital Workplace Dimensions and Productive Organizational Energy. The computed F magnitude (245.530) is higher than the table magnitude (2.46) at P = 0.05 level of significance. This means that the statistical model is statistically significant; further, the joint dimensions of the digital workplace help explain the variation in productive organizational energy. R-magnitude (0.880), which indicates a strong relationship between the variables used, and R ^2^-magnitude (0.775), which means that the dimensions of the digital workplace can explain (77.5%) of the differences in productive organizational energy. This is a reasonable proportion, indicating a high power to explain the model. The R ^2^ Adj magnitude was (0.772) indicating a good fit of the model and validating it for the analysis. As for the t-test, Table 8 indicates that all hypotheses were higher than the table magnitude (1.984) at the level of significance (0.05), indicating a statistically significant impact of most digital workplace dimensions to explain the variation occurring in PWE, yet with different levels of impact strength. Digital space had a t-magnitude of (2.581) at (β = 0.118), and the significance level was obtained as (Sig = 0.010), which means digital space moderately contributes to increases in productive organizational energy by promoting collaboration among employees and, hence, transfers frequent communication among employees. Digital aesthetics attained (t = 2.354) at (β = 0.102), and the level of significance was (Sig = 0.019), which is significant yet weak, suggesting that the aesthetic features of digital systems add somewhat to inspiring productive organizational energies by enhancing user experience and functional interaction only regarding limited weight for obtaining knowledge gains from a system design to produce work process innovations. On the other hand, the influence of digital capabilities was highly significant with a t-magnitude of (5.320) at (β = 0.277), and Sig-level 0.000, which confirms that employees’ ownership of technical capabilities as well as digital competencies contributes significantly to elevating the level of productive organizational energy and improving performance effectiveness. The highest effective dimension among the 4-dimensions was digital intelligence, as it acquired a t-magnitude = (8.332) at (β = 0.414) and significance magnitude = (Sig = 0.000), which means that the dependency of the university on analytic systems and artificial intelligence in conducting operations and coordinating information is the most useful element in triggering productive organizational energy, and maximizing the institutional performance effect. On the other hand, the findings of the multicollinearity test of (Tolerance 0.260–0.384 > 0.10) and (VIF 2.605–3.852 < 10) indicate that no problem of multicollinearity exists among all independent dimensions and indicates that the model is applicable for statistical analysis. Thus, overall, it can be concluded that the aggregate dimensions of the digital workplace do work for the development of productive organizational energy, but in varying degrees, ranging from strong impact being made by digital intelligence to, less impact being made by digital capabilities and space while limited influence was exerted by aesthetics. These findings explain that university institutions investing in smart technologies and improving employees’ ICT skills achieve better performance efficiency, stimulate the work environment, and facilitate cooperation and integration among new digital systems (intelligent networks), thereby receiving higher levels of productive organizational energy.

**Table 14.** Impact analysis of digital workplace dimensions together on productive organizational energy.

Dimensions of the digital workplace	α	β	t	Sig	R	R ^2^	(R ^2^) Adj	F	Sig	Tolerance	VIF
Digital Space	0.341	0.118	2.581	0.010	0.880	0.775	0.772	245.530	0.000	0.269	3.712
Digital Beauty		0.102	2.354	0.019						0.384	2.605
Digital Capabilities		0.277	5.320	0.000						0.260	3.852
Digital Intelligence		0.414	8.332	0.000						0.308	3.247

### 3.5 Heterotrait–Monotrait Ratio (HTMT) test – Digital workplace matrix

The Heterotrait–Monotrait ratio (HTMT) matrix showing the inter-trait correlation is provided in Table 15 for construct the digital workplace. The results demonstrate that all HTMT values are less than the acceptable cut-off point (0.90), as suggested by Henseler et al. (2015), which provides evidence of discriminant validity between the four dimensions of the digital workplace model. The coefficients are indicative of moderate relationships among dimensions, allowing for both conceptual and empirical separateness, but preserving logical unity within the construct. The HTMT analysis confirms that the digital workplace has good structural distinctiveness among its dimensions, which increases its conceptual robustness and justifies proceeding with SEM_of the main study variables.

**Table 15.** HTMT test – Digital workplace.

	DA	DC	DI	DS
**DA**	—	—	—	—
**DC**	0.812	—	—	—
**DI**	0.748	0.826	—	—
**DS**	0.735	0.785	0.711	—

### 3.6 Heterotrait–Monotrait Ratio (HTMT) test – Organizational energy matrix

Table 16 presents the HTMT matrix of the dimensions of the organizational energy construct. This test serves the purpose of testing the discriminant validity between dimensions to ensure conceptual and empirical independence. The findings reveal that all HTMT values are smaller than the predefined 0.90 criterion ( Henseler et al., 2015), which suggests that the relationships between dimensions are moderate and acceptable, thereby demonstrating a high level of distinctiveness among these dimensions. These results confirm the theoretical coherence and structural validity of the model, demonstrating that, although the three dimensions are interrelated, they are statistically and conceptually different. The two models together provide a unified and linked partition of organizational energy within the context of university-related work.

**Table 16.** HTMT test – Organizational energy.

	BE	CE	EE
BE	—	—	—
CE	0.772	—	—
EE	0.728	0.780	—

## 4. Conclusions

The research offers strong empirical insight on the strategic significance of the digital workplace for productive organizational energy in higher education institutions, using the analytical case of University of Fallujah. The studies elucidated that the digital workplace of the university is located at an average-good level of development, indicating a growing tendency within the institution for digital transformation to increase performance and operational efficiency. In terms of dimensions, digital intelligence stands out as the leading advanced and influential factor, which means that knowledge about artificial intelligence applications and digital analytics to improve decision-making, productivity, and institutional outcomes is increasing. Digital literacies and digital aesthetics were at moderate levels, which confirms that there are satisfactory computing competencies of employees to work in organizations today while training plans accompanied by user friendly interfaces are still necessary for leveraging performance. Digital utilization level scored medium, suggesting that digital collaboration between different departments is still insufficient, and large channel-based workflow integration and interaction are required.

From an inferential standpoint, the regression analysis revealed a strong and significant influence of the digital workplace on productive organizational energy (β = 0.892, R ^2^ = 0.75, F = 865.962, p < 0.001), indicating that progression in the digital milieu significantly contributes to employees’ zeal, motivation, and engagement with the organization, even though cross-sectional nature of the data may prevent definitive causal conclusions from being drown. Among the four dimensions of the digital workplace, digital intelligence had the maximum impact (β = 0.819, R ^2^ = 0.69), followed by digital capabilities (β = 0.775, R ^2^ = 0.67), digital space (β = 0.651, R ^2^ = 0.59), and digital aesthetics (β = 0.665, R ^2^ = 0.48). These results suggest that the digital workplace is not a location from which administrative tasks are supported; it is a place where emotional, cognitive, and behavioral forces converge to generate productive organizational energy. Additionally, the regression model exhibited high explanatory power and internal consistency, with no multicollinearity effect, confirming the robustness and reliability of the proposed theoretical framework.

### 4.1 Recommendations and Future Research Suggestions

The findings of this study yield a set of practical recommendations that can be useful for higher education institutions, particularly those undergoing digital transformation. These recommendations can be organized into three thematic categories: (1) Technology and Infrastructure Development, (2) Human Capital and Capability Building, and (3) Institutional Strategy and Leadership.

Category 1: Technology and Infrastructure Development

It is essential to improve digital intelligence in the universities by investing in smart technologies and using them in planning and decision-making. This is important given that digital intelligence recorded the highest impact on productive organizational energy (β = 0.819, R ^2^ = 0.69) among all digital workplace dimensions. This would confirm that the use of artificial intelligence and advanced digital analytics would contribute to activating productive organizational energies and enhancing performance efficiency in academic institutions, as desired. Improving the digital space environment by expanding the use of collaborative digital platforms and integrating administrative and academic units is strongly recommended. Digital space recorded a mean score of (M = 3.297), the lowest among all digital workplace dimensions, suggesting that digital collaboration between different departments remains insufficient and that large channel-based workflow integration and interaction are still required.

Attention should be paid to digital aesthetics by designing interactive and attractive digital interfaces that contribute to increasing job satisfaction and motivating employees to interact with electronic systems. Digital aesthetics recorded a mean score of (M = 3.386) at a medium level, indicating that further efforts are needed to better infuse interface design and user experience improvements for a more meaningful impact on increasing work efficiency.

Category 2: Human Capital and Capability Building

Developing the digital capabilities of employees through continuous training and technical support to develop their skills in dealing with digital tools and platforms is strongly recommended. Digital capabilities recorded a mean score of (M = 3.294) at a medium level, representing a modest level of digital proficiency among employees, which underscores the importance of extending training and skills programs to improve the technical efficiency necessary to handle digitalization.

In data-poor environments, the use of open-source AI toolkits in combination with regional digital training and incremental capacity building facilitates optimal digital intelligence and sustainable institutional performance. This recommendation is particularly relevant to the Iraqi higher education context, where infrastructure limitations and uneven digital proficiency among employees may constrain the full expression of digital workplace benefits.

Category 3: Institutional Strategy and Leadership

Implementing a holistic digital workplace model, considered through the four dimensions of the digital workspace, which are digital space, digital aesthetics, digital capabilities, and digital intelligence, with a single institutional vision is essential to drive organizational performance and create an inspiring and efficient working environment. The regression analysis confirmed that the collective dimensions of the digital workplace explain 77.5% of the differences in productive organizational energy (R ^2^ = 0.775, F = 245.530, p < 0.001). This would underscore the importance of an integrated rather than fragmented approach to digital transformation.

The relative cultural context of Iraq, which is characterized by a hierarchical structure and collectiveness, moderates the effect of the digital workplace on organizational energy. Encouraging leadership cultivation, digital trust, and culturally embedded partnerships can foster employees’ emotional and cognitive engagement. This recommendation is directed at senior university leadership and policy makers who are best positioned to shape the institutional culture and create an environment conducive to digital transformation and productive organizational energy.

While this study provides valuable empirical insights into the role of the digital workplace in enhancing productive organizational energy, several limitations should be acknowledged to guide future research and to contextualize the generalizability of the findings. For example, the study was conducted exclusively at the University of Fallujah, a single higher education institution located in Iraq. Although this context provides a focused and internally consistent analytical setting, it limits the generalizability of the findings to other universities, academic institutions, or organizational sectors with different structural, cultural, or technological characteristics. Future studies are encouraged to replicate this research across multiple universities or across different types of organizations to enhance the external validity of the proposed theoretical framework. Multi-site and cross-sectoral replication studies would significantly enhance the external validity of the findings and allow researchers to examine whether the relative ranking of digital workplace dimensions in terms of their impact on productive organizational energy — with digital intelligence at the top, followed by digital capabilities, digital aesthetics, and digital space — holds consistently across different institutional and cultural settings. Therefore, future research replicating this framework across multiple institutions and cultural contexts would significantly strengthen the external validity of these findings and advance the broader digital transformation literature in higher education.

Second, the sample composition reflects a notable gender imbalance, with male participants constituting 80.3% of the total sample of 290 respondents, while female participants represented only 19.7%. As acknowledged in the paper, this disproportion may be attributed to the largely male functional and academic organizational structure of the university. This imbalance may limit the extent to which findings can be generalized across gender groups, and future research should endeavor to achieve more balanced gender representation to allow for comparative gender-based analysis of digital workplace perceptions and organizational energy.

Third, the study relied on a self-reported questionnaire as the primary data collection instrument. Although the instrument demonstrated strong reliability and validity indicators including Cronbach’s alpha values exceeding 0.70, composite reliability values ranging between 0.874 and 0.937, and AVE values exceeding 0.50 across all dimensions’ self-reported data are inherently susceptible to common method bias and social desirability bias, which may influence the accuracy of responses regarding digital workplace perceptions and organizational energy levels.

Fourth, even though the research design of descriptive-analytical that is used in this study is feasible for examining relationships between variables at a specific point in time, it does not allow for causal inference or the tracking of changes in the evolution of the digital work environment and organizational capacity over time. Research designs would be more appropriate for monitoring the dynamic evolution of these variables within academic institutions undergoing digital transformation. Future research employing longitudinal designs, experimental methods, or structural equation modeling with panel data would be better able to identify the causal mechanisms underlying the correlations identified in this study and track how changes in the evolution of the digital work environment over time relate to corresponding changes in productive organizational capacity within academic institutions.

### Ethical considerations

This study involved human participants and was conducted in accordance with accepted ethical research standards and the principles outlined in the Declaration of Helsinki. Ethical approval was obtained from the Scientific Research Ethics Committee, University of Fallujah, Iraq (Approval No. HOF.HUM.2025.001). Written informed consent was obtained from all participants prior to their participation. All participants were informed about the purpose of the study, the voluntary nature of their participation, their right to withdraw at any time without consequences, and the confidentiality of their data.

## Informed consent

Written informed consent was obtained from all individual participants prior to their participation in the study. All participants were informed about the purpose of the research, their right to withdraw at any time, and the confidentiality of their data.

## Data availability

The data supporting the findings of this study are openly available in Zenodo at: https://doi.org/10.5281/zenodo.18290375, Laith Emad, A., & Lateef Khalaf, Y. (2026). The Role of the Digital Workplace in Enhancing Productive Organizational Energy: An Analytical Study at the University of Fallujah. Zenodo.

The data are available under the terms of the Creative Commons Attribution 4.0 International license (CC-BY 4.0).

### Reporting guidelines

This study is observational survey-based research and follows the STROBE reporting guidelines. No CONSORT or ARRIVE checklists are required as the study does not involve clinical trials or animal experiments.
