# Supplementary material for: The Role of the Digital Workplace in Enhancing Productive Organizational Energy: An Analytical Study at the University of Fallujah
Source: F1000Res. 2026 May 19;15:234. Originally published 2026 Feb 11. [Version 2] doi: 10.12688/f1000research.174088.2 (PMC13220924; doi:10.12688/f1000research.174088.2)
Supplement: Peer review history

# Reviewer report: Reviewer response for version 2

**DOI:** 10.5256/f1000research.197968.r486398

**Published:** 2026-05-25

**Authors:** Aleksy Kwilinski, Maciej Kardas

**Affiliations:** 1 The London Academy of Science and Business, London, UK

Compared with the original submission, the manuscript has been substantially improved. The revised version provides a clearer theoretical framework, a better explanation of the research model and hypotheses, and a more detailed methodological description.

**Clarity of presentation and use of literature**

 The manuscript is generally clearly structured and the authors cite relevant literature related to digital workplace transformation and productive organizational energy. In the revised version, the literature review has been strengthened by more explicitly linking the four digital workplace dimensions with productive organizational energy. The theoretical justification for the proposed research model is now clearer and better connected to previous studies.

 Some minor language and stylistic issues remain, and further English proofreading would still be beneficial. However, these issues do not substantially undermine the scientific contribution of the manuscript.

**Study design and technical soundness**

 The overall research design, based on survey data and statistical analysis, is appropriate for the research question. The use of reliability analysis, confirmatory factor analysis, HTMT assessment, and regression analysis is suitable for evaluating the proposed constructs and testing the hypothesized relationships.

 The authors have also more clearly acknowledged the limitation related to data collected from a single institution and discussed its effect on the generalizability of the findings. This improves the balance and transparency of the manuscript.

**Methods and reproducibility**

 The revised manuscript provides additional methodological details, including more information about the questionnaire, measurement scales, reliability indicators, composite reliability, AVE, and discriminant validity. These additions improve the transparency and reproducibility of the study.

 However, the study still relies on a single self-reported data source collected at one point in time. This may increase the risk of common method bias and inflated associations between variables. This limitation should be kept in mind when interpreting the strength of the reported relationships.

**Statistical analysis and interpretation**

 The statistical methods used in the paper are appropriate for the proposed research model. The authors report reliability measures, validity indicators, and regression results that support the main findings.

 The interpretation of the results is now more cautious than in the original version. In particular, the revised manuscript better acknowledges that, due to the cross-sectional nature of the data, the findings should be understood mainly as statistical associations rather than definitive causal relationships.

Is the work clearly and accurately presented and does it cite the current literature?

Partly

If applicable, is the statistical analysis and its interpretation appropriate?

Partly

Are all the source data underlying the results available to ensure full reproducibility?

Yes

Is the study design appropriate and is the work technically sound?

Partly

Are the conclusions drawn adequately supported by the results?

Partly

Are sufficient details of methods and analysis provided to allow replication by others?

Partly

Reviewer Expertise:

Management science and quality management

We confirm that we have read this submission and believe that we have an appropriate level of expertise to confirm that it is of an acceptable scientific standard.

# Reviewer report: Reviewer response for version 1

**DOI:** 10.5256/f1000research.191963.r463594

**Published:** 2026-03-14

**Authors:** Aleksy Kwilinski, Maciej Kardas

**Affiliations:** 1 The London Academy of Science and Business, London, UK

The manuscript investigates the relationship between the digital workplace and productive organizational energy in a higher education context. The authors examine four dimensions of the digital workplace - digital space, digital aesthetics, digital capabilities, and digital intelligence -and analyse their relationship with three components of productive organizational energy: emotional, cognitive, and behavioral energy.

 The study is based on survey data collected from 290 respondents affiliated with the University of Fallujah. The authors apply statistical analysis using SPSS and SmartPLS, including reliability analysis, confirmatory factor analysis, and regression analysis. The results indicate a positive relationship between digital workplace dimensions and productive organizational energy.

 The topic is relevant, particularly in the context of digital transformation and organizational performance.

**Clarity of presentation and use of literature**

- The manuscript is generally clearly structured and the authors cite relevant literature related to digital workplace transformation and organizational energy. However, the literature review is largely descriptive and could be strengthened by more clearly explaining how previous studies support the proposed research model and hypotheses.
- To improve this section, the authors could further develop the theoretical discussion linking digital workplace dimensions with productive organizational energy.

**Study design and technical soundness**

- The overall research design using survey data and statistical analysis is appropriate for the research question. Techniques such as reliability analysis and confirmatory factor analysis are suitable for evaluating the proposed constructs. However, the study is based on data collected from a single institution. The authors should acknowledge this limitation more clearly and discuss how it may affect the generalizability of the findings.

**Methods and reproducibility**

- The manuscript presents the main statistical procedures used in the analysis and reports reliability indicators for the constructs. However, providing additional details about the survey instrument would improve transparency. Including the questionnaire items or measurement scales as supplementary material would make it easier for other researchers to replicate the study.

**Statistical analysis and interpretation**

- The statistical methods used in the paper appear appropriate for the proposed research model. The authors report reliability measures and regression results that support the main findings. However, the interpretation of the results should remain cautious. Since the study relies on cross-sectional survey data, the discussion should focus on associations between variables rather than implying strong causal relationships.

Is the work clearly and accurately presented and does it cite the current literature?

Partly

If applicable, is the statistical analysis and its interpretation appropriate?

Partly

Are all the source data underlying the results available to ensure full reproducibility?

Yes

Is the study design appropriate and is the work technically sound?

Partly

Are the conclusions drawn adequately supported by the results?

Partly

Are sufficient details of methods and analysis provided to allow replication by others?

Partly

Reviewer Expertise:

Management science and quality management

We confirm that we have read this submission and believe that we have an appropriate level of expertise to confirm that it is of an acceptable scientific standard, however we have significant reservations, as outlined above.

# Response

**Published:** 2026-03-20

**Authors:** amna alhashimi

**Affiliations:** public administration, university of fallujah, iraq, baghdad, Iraq

The authors would like to thank the editor and the reviewers for their time and effort.  We would also like to thank the editor for considering resubmitting our manuscript and giving us the opportunity to address the reviewers’ comments. The authors would like to thank the reviewer for the detailed and constructive comments on improving our manuscript. Their insightful feedback has significantly improved the paper. We would like to highlight that we have undertaken a comprehensive set of modifications and refinements in the revised version of our manuscript. These revisions have been carefully addressed to ensure that our study fully aligns with the required standards and expectations. The manuscript has been revised based on the reviewer comments, and the suggested amendments, together with our responses, are detailed in the following paragraphs. Specifically, in the following paragraphs, we provide our responses to the issues raised by the reviewers.

 1. The study is based on survey data collected from 290 respondents affiliated with the University of Fallujah. The authors apply statistical analysis using SPSS and SmartPLS, including reliability analysis, confirmatory factor analysis, and regression analysis. The results indicate a positive relationship between digital workplace dimensions and productive organizational energy.

**Response: ** Thank you very much for providing us with the feedback on the original version of the paper. We sincerely thank the reviewer for the valuable feedback and thoughtful suggestions. The authors would like to thank you very much for reading our manuscript and provide the valuable feedback.

 2. Clearly The manuscript investigates the relationship between the digital workplace and productive organizational energy in a higher education context. The authors examine four dimensions of the digital workplace - digital space, digital aesthetics, digital capabilities, and digital intelligence -and analyse their relationship with three components of productive organizational energy: emotional, cognitive, and behavioural energy.

**Response: **The authors would like to thank you very much for reading our manuscript and providing the valuable feedback. We thank the reviewer for the great feedback and are grateful for the reviewer's comments that increased the quality and readability of the manuscript. Your insightful comments and constructive feedback have been invaluable in enhancing the quality and clarity of our work.

 3. The topic is relevant, particularly in the context of digital transformation and organizational performance.

**Response:** The authors would like to thank you very much for reading our manuscript and providing the valuable feedback. We thank the reviewer for the great feedback and are grateful for the reviewer's comments that increased the quality and readability of the manuscript. Your insightful comments and constructive feedback have been invaluable in enhancing the quality and clarity of our work.  

 4. The manuscript is generally clearly structured and the authors cite relevant literature related to digital workplace transformation and organizational energy. However, the literature review is largely descriptive and could be strengthened by more clearly explaining how previous studies support the proposed research model and hypotheses. To improve this section, the authors could further develop the theoretical discussion linking digital workplace dimensions with productive organizational energy.

**Response:** The author would like to thank the reviewer for this insightful and constructive observation. We would like to pointed out that the literature review required strengthening to more explicitly establish the theoretical linkages between the digital workplace dimensions and productive organizational energy, and to more clearly justify the proposed research model and its hypotheses. Based on the reviewer comment, we have provided a more theoretical discussion and explanation in the Introduction section to accommodate the reviewer comments. Again, we would like to thank the reviewer for the feedback.

 5. Study design and technical soundness. The overall research design using survey data and statistical analysis is appropriate for the research question. Techniques such as reliability analysis and confirmatory factor analysis are suitable for evaluating the proposed constructs. However, the study is based on data collected from a single institution. The authors should acknowledge this limitation more clearly and discuss how it may affect the generalizability of the findings.

**Response:** We sincerely thank the reviewer for this important observation. We fully agree that the single-institution nature of the data collection represents a significant scope boundary that requires more explicit acknowledgement and deeper discussion in terms of its specific implications for the generalizability of the findings. While we had briefly acknowledged this point in the Limitations section, we recognize that the reviewer rightly calls for a more thorough and nuanced discussion of how this methodological characteristic may affect the external validity and transferability of the study's conclusions. Accordingly, we have substantially expanded this discussion both within the Limitations section and within the Conclusions section.

 6. Methods and reproducibility. The manuscript presents the main statistical procedures used in the analysis and report's reliability indicators for the constructs. However, providing additional details about the survey instrument would improve transparency. Including the questionnaire items or measurement scales as supplementary material would make it easier for other researchers to replicate the study.

**Response:** The authors would like to thank you very much for reading our manuscript and provide the valuable feedback. We really appreciate your time and effort in reading our manuscript. Your thoughtful insights and encouraging remarks have played a key role in improving both the clarity and quality of our work. Accordingly, we have provided the questionnaire in the manuscript. The instrument comprises two main variables: the digital workplace variable, measured through 18 items distributed across four dimensions — digital space (DS1–DS5), digital aesthetics (DA1–DA4), digital capabilities (DC1–DC4), and digital intelligence (DI1–DI5) — and the productive organizational energy variable, measured through 17 items distributed across three dimensions — behavioural energy (BE1–BE5), cognitive energy (CE1–CE6), and emotional energy (EE1–EE6). All items were rated on a five-point Likert scale. The factor loadings for all questionnaire items are already reported in Tables 10 and 12 of the manuscript, confirming that all items exceeded the minimum acceptable factor loading threshold of 0.50, with values ranging between 0.685 and 0.872 for the digital workplace variable and between 0.658 and 0.924 for the productive organizational energy variable. We sincerely thank the reviewer for this constructive suggestion regarding transparency and replicability. We fully agree that providing additional details about the survey instrument enhances the transparency of the research process and facilitates replication by future researchers.

 7. Statistical analysis and interpretation. The statistical methods used in the paper appear appropriate for the proposed research model. The authors report reliability measures and regression results that support the main findings. However, the interpretation of the results should remain cautious. Since the study relies on cross-sectional survey data, the discussion should focus on associations between variables rather than implying strong causal relationships.

**Response:** The authors would like to thank you very much for reading our manuscript and provide the valuable feedback. We really appreciate your time and effort in reading our manuscript. Your thoughtful insights and encouraging remarks have played a key role in improving both the clarity and quality of our work. We are especially thankful for your constructive feedback, which has greatly contributed to strengthening the overall presentation and depth of our study. We have carefully revised the manuscript to ensure that the interpretation of the results remains cautious. Specifically, causal language has been replaced with association-based expressions where appropriate. In addition, a clarification has been added in the discussion section to the revised version of the paper to emphasize that the findings are based on cross-sectional data and therefore indicate associations rather than causal relationships. Please see the red text in the revised version of the paper.

 Once again, we sincerely appreciate your valuable insights, which have significantly improved the quality of our research. We are confident that the revised manuscript now fully meets the required standards. Thank you for your constructive feedback and for your essential role in the review process.

# Reviewer report: Reviewer response for version 1

**DOI:** 10.5256/f1000research.191963.r458737

**Published:** 2026-03-11

**Authors:** Silvia Farias-Gaytan

**Affiliations:** 1 Tecnologico de Monterrey, Monterrey, Mexico

The title expresses the aim of the manuscript and includes keywords from its main constructs.

 The Abstract is structured with the aim, methods, results and conclusions of the research presenting the necessary information.

 The Introduction includes recent and relevant literature about the constructs and context of the research and identifies the gap of how digital transformation impacts employees’ emotional, cognitive, and behavioral engagement. The hypotheses analyzed in the Results and discussion section can be mentioned in the Introduction to improve structure and coherence throughout the paper.

 The research methodology can be strengthened by including references to support the analytical method chosen. The Methods section is missing information on the instrument used to gather information from the participants. It doesn’t indicate if the instrument is original or taken from another research.

 The Results and discussion section describe the statistical analysis for each variable and includes references to previous research to support the analysis.

 The Conclusions identify the contribution provided by the analysis and its relevance to organizations; however, there is no Limitations which is important to include so that it serves other researchers interested in replicating the study.

 The Recommendations section although it consists of 7 statements, could be useful to identify some categories to group them and help highlight the stakeholders.

Is the work clearly and accurately presented and does it cite the current literature?

Yes

If applicable, is the statistical analysis and its interpretation appropriate?

I cannot comment. A qualified statistician is required.

Are all the source data underlying the results available to ensure full reproducibility?

Yes

Is the study design appropriate and is the work technically sound?

Yes

Are the conclusions drawn adequately supported by the results?

Yes

Are sufficient details of methods and analysis provided to allow replication by others?

Partly

Reviewer Expertise:

educational innovation, digital education, digital transformation in education, learning spaces

I confirm that I have read this submission and believe that I have an appropriate level of expertise to confirm that it is of an acceptable scientific standard.

# Response

**Published:** 2026-03-20

**Authors:** amna alhashimi

**Affiliations:** public administration, university of fallujah, iraq, baghdad, Iraq

**The Role of the Digital Workplace in Enhancing Productive Organizational Energy: An Analytical Study at the University of Fallujah**

**Revision**

**All additions and changes to the original manuscript are highlighted in new revision.**

**Original Article Title: **The Role of the Digital Workplace in Enhancing Productive Organizational Energy: An Analytical Study at the University of Fallujah

 The authors would like to thank the editor and the reviewers for their time and effort.  We would also like to thank the editor for considering resubmitting our manuscript and giving us the opportunity to address the reviewers’ comments. The authors would like to thank the reviewer for the detailed and constructive comments on improving our manuscript. Their insightful feedback has significantly improved the paper. We would like to highlight that we have undertaken a comprehensive set of modifications and refinements in the revised version of our manuscript. These revisions have been carefully addressed to ensure that our study fully aligns with the required standards and expectations. The manuscript has been revised based on the reviewer comments, and the suggested amendments, together with our responses, are detailed in the following paragraphs. Specifically, in the following paragraphs, we provide our responses to the issues raised by the reviewers.

**Response to Reviewers**

**Reviewer#1 Comments**

  

1. The title expresses the aim of the manuscript and includes keywords from its main constructs.

**Response:** We would like to thank you very much for reading our manuscript and provide the valuable feedback. We would also like to thank you for the positive feedback. The authors would also like to thank the reviewer for the great feedback and are grateful for the reviewer's comments that increased the quality of the manuscript. Your insightful comments and constructive feedback have been invaluable in enhancing the quality and clarity of our work.

 2. The Abstract is structured with the aim, methods, results and conclusions of the research presenting the necessary information.

**Response:** The authors would like to thank the reviewer very much for reading our manuscript and provide the positive feedback. The authors would also like to thank the reviewer for the great reviewer's comments that increased the quality of the manuscript. Your insightful comments and constructive feedback have been invaluable in enhancing the quality and clarity of our work.

 3. The Introduction includes recent and relevant literature about the constructs and context of the research and identifies the gap of how digital transformation impacts employees’ emotional, cognitive, and behavioral engagement. The hypotheses analyzed in the Results and discussion section can be mentioned in the Introduction to improve structure and coherence throughout the paper.

**Response:** We would like to thank the reviewer for the great feedback and are grateful for the reviewer's comments that increased the quality of the manuscript. Your insightful comments and constructive feedback have been invaluable in enhancing the quality and clarity of our work. We have addressed this issue in the revised version of the paper. In response to this comment, we have added a discussion highlighting the hypotheses analyzed in the Introduction to improve structure and coherence throughout the paper.

 4. The research methodology can be strengthened by including references to support the analytical method chosen. The Methods section is missing information on the instrument used to gather information from the participants. It doesn’t indicate if the instrument is original or taken from another research.

**Response:** We would like to thank the reviewer for the great feedback and are grateful for the reviewer's comments that increased the quality of the manuscript. Your insightful comments and constructive feedback have been invaluable in enhancing the quality and clarity of our work.  In response to this comment, we have addressed this issue in the revised version of the paper.  

 5. The Results and discussion section describe the statistical analysis for each variable and includes references to previous research to support the analysis.

**Response:** We would like to thank the reviewer for the great feedback and are grateful for the reviewer's comments that increased the quality of the manuscript. Your insightful comments and constructive feedback have been invaluable in enhancing the quality and clarity of our work. We have addressed this issue in the revised version of the paper. In response to this comment regarding the Results and Discussion section. We are pleased in the revised version of the paper that the statistical analysis and its alignment with previous research literature were found to be adequate and well-supported throughout the paper.

 6. The Conclusions identify the contribution provided by the analysis and its relevance to organizations; however, there is no Limitations which is important to include so that it serves other researchers interested in replicating the study

**Response: **The authors would like to thank you very much for reading our manuscript and providing the valuable feedback. We are grateful for your meticulous assessment and for providing recommendations that have significantly contributed to refining our study. In response, we have considered the reviewer comment and improve the paper accordingly by adding the limitations to the revised version of the paper.

 7. The Recommendations section although it consists of 7 statements, could be useful to identify some categories to group them and help highlight the stakeholders.

**Response:** We would like to thank you very much for reading our manuscript and provide the valuable feedback. We would also like to thank you for the positive feedback. The authors would also like to thank the reviewer for the great feedback and are grateful for the reviewer's comments that increased the quality of the manuscript. Your insightful comments and constructive feedback have been invaluable in enhancing the quality and clarity of our work. We have addressed this issue in the revised version of the paper

 Once again, we sincerely appreciate your valuable insights, which have significantly improved the quality of our research. We are confident that the revised manuscript now fully meets the required standards. Thank you for your constructive feedback and for your essential role in the review process.
